# Pyrolysis of Polypropylene and Nitrile PPE Waste: Insights into Oil Composition, Kinetics, and Steam Cracker Integration

**DOI:** 10.3390/molecules30163351

**Published:** 2025-08-12

**Authors:** Ross Baird, Raffaella Ocone, Aimaro Sanna

**Affiliations:** 1 Institute of Mechanical, Process and Energy Engineering, School of Engineering and Physical Sciences, Heriot-Watt University, Edinburgh EH14 4AS, UK; rb56@hw.ac.uk; 2 Institute of GeoEnergy Engineering, School of Energy, Geoscience, Infrastructure and Society, Heriot-Watt University, Edinburgh EH14 4AS, UK; r.ocone@hw.ac.uk

**Keywords:** masks, gloves, pyrolysis, product analysis, kinetic analysis, steam cracking

## Abstract

In this study, non-isothermal pyrolysis of a mixture of disposable surgical face masks (FMs) and nitrile gloves (NGs) was conducted, using a heating rate of 100 °C/min, N_2_ flowrate of 100 mL/min, and temperatures between 500 and 800 °C. Condensable product yield peaked at 600 °C (76.9 wt.%), with gas yields rising to 31.0 wt.%, at 800 °C. GC-MS of the condensable product confirmed the presence of aliphatic compounds (>90%), while hydrogen, methane, and ethylene dominated the gas composition. At 600 °C, gasoline (C_4_ to C_12_)-, diesel (C_13_ to C_20_)-, motor oil (C_21_ to C_35_)-, and heavy hydrocarbon (C_35+_)-range compounds accounted for 23.7, 46.7, 12.5, and 17.1%, of the condensable product, respectively. Using model-free methods, the average activation energy and pre-exponential factor were found to be 309.7 ± 2.4 kJ/mol and 2.5 ± 3.4 × 10^25^ s^−1^, respectively, while a 2-dimensional diffusion mechanism was determined. Scale-up runs confirmed high yields of condensable product (60–70%), with comparable composition to that obtained from lab-scale tests. The pyrolysis oil exceeds acceptable oxygen, nitrogen, chlorine, and fluorine levels for industrial steam crackers—needing pre-treatment—while other contaminants like sulphur and metals could be managed through mild blending. In summary, this work offers a sustainable approach to address the environmental concerns surrounding disposable FMs and NGs.

## 1. Introduction

Since commercial production began in the 1950s, global dependence on plastics has risen exponentially [1]. Currently, plastics are used extensively due to their versatility, simplicity, and low production costs [2]. As of 2023, global plastic production is estimated at around 400 million tonnes and is forecast to rise to over one billion tonnes by 2060 [3]. Fuelling this increase, in part, is the use of plastic products with a short service life such as Personal Protective Equipment (PPE), which are recommended to be disposed of after a sole use [4]. Today, less than 10% of all plastic waste is recycled, with the majority (∼50%) still sent to landfill [5]. PPE waste, such as nitrile gloves (NG) and face masks (FM), is highly unsuitable for landfilling due to its potential to cause microplastic pollution in waterways, risk of biohazard contamination, and non-biodegradable nature. Additionally, landfilling is rapidly becoming a less-than-desirable method of plastic waste treatment, due to the known harm its practice has on the surrounding environment [6].

Of the plastic recycling methods currently in use, mechanical recycling remains the most prevalent. However, PPE waste is usually a heterogeneous mixture, limiting its suitability for extrusion due to potential incompatibility between different plastics and the challenges around efficient separation [7]. For these reasons, thermal or thermochemical methods are considered to show the most promise for economical and sustainable upcycling of plastic wastes, including PPE waste [8]. Among these, incineration is currently employed for disposal of used PPE waste, but has the drawback of substantial air pollution concerns [9], especially at temperatures greater than 1000 °C, which can lead to the formation dioxins and furans, which are both highly toxic [10]. As a result, flue gas treatment is imperative, which increases the overall cost and complexity of the process [11]. Due to the unsuitability of the more conventional methods of plastic recycling mentioned, attention has turned to pyrolysis [12], a thermochemical process which involves the thermal degradation of organic material at moderate temperatures, in the absence of oxygen, to generate value-added products, such as crude bio-oil [13]. Pyrolysis has several advantages, including the capacity to process the plastic mixtures likely to be present in PPE waste [14], improved energy efficiency when compared with incineration, and reduced pollution due to the anaerobic conditions employed [15].

There are different types of pyrolysis: slow/conventional, intermediate, fast, and flash [16]. Fast pyrolysis involves rapid thermal degradation of organic material using high heating rates (10–200 °C/s), moderate temperatures (400–600 °C), and low residence times (<5 s), to limit secondary cracking reactions, and prioritise liquid bio-oil production. Bio-oil yields as high as 95 wt.% are possible, depending upon the type of plastic used [9]. The liquid oil is usually a mixture of hundreds of different compounds, and the physical properties vary depending on the operating parameters used. Intermediate pyrolysis focuses on the production of liquid fuels, due to the moderate temperatures (500–600 °C) and higher vapour residence times (10 to 20 s) it employs. However, fast (and flash) pyrolysis requires very small and homogeneous particles to achieve fast heat and mass transfer, and, for heterogeneous waste as PPE waste, intermediate pyrolysis has proven to be more viable [17].

Two of the most common types of plastic used in the PPE industry are polypropylene (PP) and nitrile rubber. Despite this, there are a limited number of studies which have conducted intermediate pyrolysis of PPE waste mixtures, determination of kinetic parameters, and characterisation of the major products. Aragaw and Mekonnen [2] conducted TGA and slow pyrolysis on surgical masks and gloves. Heating at 15 °C/min to 600 °C, they used 300 g total feed in a steel reactor at 400 °C for 1 h. Vapours were condensed at 30–35 °C, yielding 75% oil/wax, 10% char, and 15% gas. They found that PPE waste was convertible to fuel via pyrolysis but noted variable results, recommending further testing with catalysts and temperature variation. Khaskhachikh et al. [18] analysed medical waste pyrolysis using TGA at a 10 °C/min rate to determine kinetics and product distribution at 600 °C. Polyolefins and nitrile gloves decomposed mostly between 400–500 °C. Polyolefins yielded up to 90% liquid tar, rich in organics like alkanes and aromatics. Gloves lost 80 wt.% mass, producing HCN, acrylonitrile, and ammonia, with 65 wt.% tar and 13 wt.% gas. Activation energies ranged from 277–372 kJ/mol for plastics, and 191 kJ/mol for gloves. At 600 °C, tar was the main product (50 wt.%), while gas yield rose with temperature, composed mainly of CO_2_, CO, and C_2_H_6_. Carbon shifted from char (at 400 °C) to tar (at 600 °C), which also retained most hydrogen and oxygen. Gerasimov et al. [19] performed TGA (ambient–900 °C, 10 °C/min, 50 mL/min N_2_) and slow pyrolysis of mixed medical waste in a fixed-bed reactor. Char yield dropped from ~62 wt.% at 400 °C to 12 wt.% at 650 °C. Pyrolysis oil increased with temperature, peaking at 50 wt.% at 600 °C, then declined due to secondary cracking. Gas yield rose steadily, reaching ~18–19 wt.% at 650 °C. At 400 °C, gas was mainly CO_2_ (65.6 wt.%), CO (24.2 wt.%), and CH_4_ (8.0 wt.%). Based on the literature above, the exact chemical composition and physical properties of the condensable products from pyrolysing a mixture of face masks (FMs) and nitrile gloves (NGs) under intermediate pyrolysis conditions—such as those in rotary kiln or auger reactors—remain unknown. There is limited information on the presence of halogens, oxygenates, nitrogenates, and metals needed to inform downstream processing. To enhance the circular material loop, PPE pyrolysis oil can optimally be utilised in oil refineries to produce polymers through steam cracking. To enable this, the oil must be compatible with naphtha or transformed firstly into another product with suitable volatility that meets the specific requirements of the chosen steam cracker. This can involve an intermediate step to reduce the chain length of C_16_+ straight chain hydrocarbons to that of paraffinic naphtha via hydrotreatment [20,21]. A detailed assessment of the contaminants and hydrocarbon structure of these oils is crucial to ascertain the economic feasibility of using them as a feed for steam cracking, as well as design tailored pretreatment steps to ensure they meet the entry specifications. Face masks and nitrile gloves both contain potential contaminants that can impact the performance of catalysts (through poisoning or fouling) or negatively impact the steam cracking unit by other means, such as corrosion or reducing product quality. Metals, such as Ca and Na, are known to cause harm by accumulating on the catalyst surface, preventing access to active sites (catalyst fouling), while Ni and Fe (among others) adsorb onto and block the active sites, permanently decreasing their functionality (catalyst poisoning). The existing literature provides little information on these aspects. As a result, there is a great deal of uncertainty into the potential applications for which PPE waste pyrolysis oils may be suited [22]. Therefore, a full characterisation of the condensable product obtained from pyrolysis of FMs and NGs is required, to ascertain its suitability for potential application in steam crackers. This study provides a full and detailed characterisation of the products obtained from pyrolysis of a 50:50 mass mixture of FMs and NGs and assesses the product distribution at a range of temperatures between 500 and 800 °C under intermediate pyrolysis conditions. Furthermore, the kinetic parameters of the process were determined to inform on process scale-up.

In summary, this study contributes to bridging this gap by providing critical insights into the upcycling of PPE waste pyrolysis oils. It identifies the key parameters required for successful integration into steam crackers and advances the understanding of their potential role within conventional petroleum-refining infrastructure.

## 2. Results

### 2.1. Feedstock Characterisation

**Figure 1:**
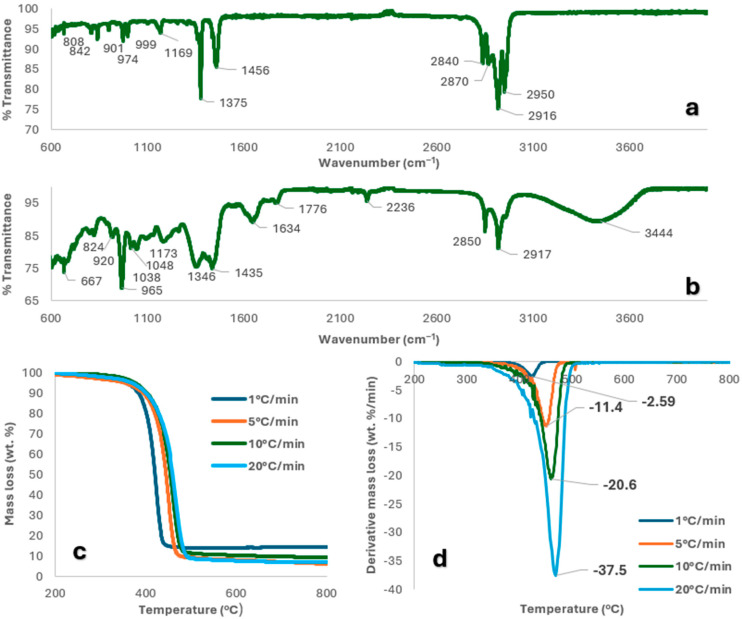
(**a**) Mask offcut and (**b**) NG FTIR spectra, and (**c**,**d**) TG and DTG curves for PPE mixture.

Figure 1a,b show the FTIR spectra obtained for the mask offcuts and NGs. For the NGs, the broad peak at 3444 cm^−1^ indicated -OH bond vibrations. The small, sharp peak at 2236 cm^−1^ was attributed to the nitrile group, and the sharp peaks at 1634 cm^−1^ to the C=C in the butadiene groups of the polymer chain [23]. C-H symmetric, asymmetric, and deformation vibrations were confirmed by the peaks at 2917, 2850, 1435, 1346, and 965 cm^−1^ [24]. The FTIR spectrum for the mask offcuts confirmed the presence of both PP and PE, but with a clear predominance of PP. Peaks at 2950, 2916, and 2840 cm^−1^ indicated C-H bond stretching. CH_2_ deformation and symmetrical CH_3_ deformation were attributed to the peaks at 1456 and 1375 cm^−1^, respectively. The C-C backbone of the polymer chain was shown by the peak at 1169 cm^−1^, with peaks at 999, 974, and 842 cm^−1^ indicating the isotactic nature of the PP [25]. The proximate analysis of mask offcuts and NGs presented in Table 1 show that both feeds have very little moisture content or fixed carbon. However, the mask offcuts are 97.52 wt.% volatile matter, compared with the 84.74 wt.% of NGs. Greater volatile matter and less ash has been previously proven to improve the ease of ignition and enhance the heating value. Lower ash has also been linked to minimising fouling and slagging in boilers [26]. Ultimate analysis showed that the mask offcuts consist mostly of carbon and hydrogen. NGs, on the other hand, had a significant concentration of both nitrogen (due to the acrylonitrile group of the nitrile repeating unit), and oxygen, assumed to be due to the presence of O-containing additives used during the manufacturing process, such as the plasticiser bis(2-ethylhexyl) phthalate and 2-ethylhexyl benzoate [27]. As shown, due to the higher ash, oxygen, and nitrogen content of the NGs, it has an HHV that is notably inferior to that of the mask offcuts.

Figure 1c,d portray the TGA and DTG profiles of the PPE mixture between 30 and 900 °C obtained using all heating rates. Between 150 and 600 °C, the main thermal degradation stage occurs, with a loss of 91.86 wt.% observed, due to the devolatilisation of light organic compounds and heavier carbon-containing organic compounds.

From the DTG plot, the highest rate of mass loss occurred between 422.75 and 469 °C and increased with a rise in the heating rate used. In the last stage, between 600 and 900 °C, an additional mass loss of approximately 1.79 wt.% was observed. This is consistent with the literature, in which PP and PE were found to thermally decompose between 435 and 500 °C and 9.5 wt.% residual mass [28], while NGs in the range 400–500 °C, leaving 20 wt.% residual mass [18].

### 2.2. Thermal Pyrolysis of Mask Offcuts and Nitrile Gloves

**Figure 2:**
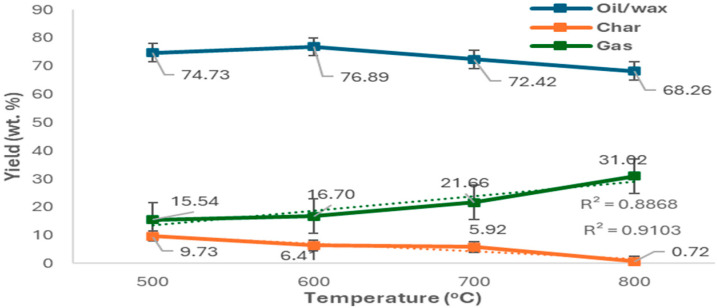
Product yields obtained from PPE mixture pyrolysis at different temperatures.

**Figure 3:**
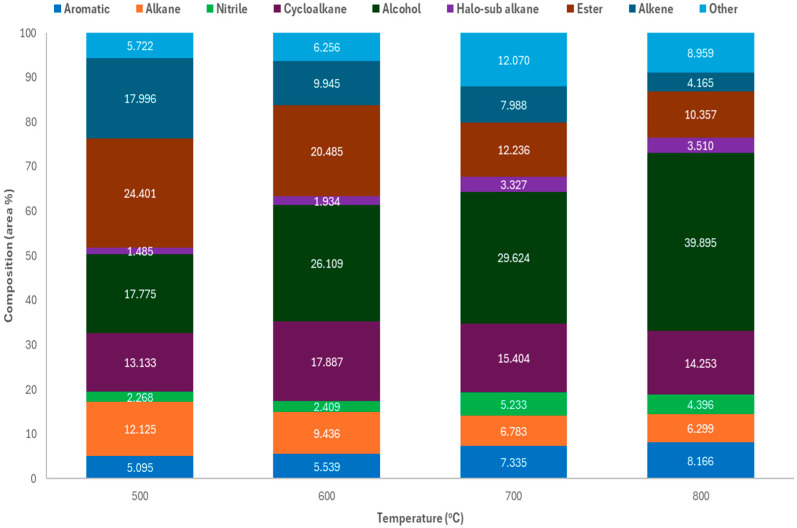
Comparison of condensable product obtained at different temperatures.

To assess product distribution by temperature, non-catalytic pyrolysis of PPE was performed at 500–800 °C. Peak condensable yield (76.89 wt.%) occurred at 600 °C as visible in Figure 2. Gas yield rose with temperature, while char stayed below 10 wt.% and declined. This suggests increased cracking at higher temperatures. Results matched Gu et al. [29], who reported 71%, 8%, and 21% yields for oil, char, and gas at 550 °C. The condensable products across all temperatures (Figure 3 and Table S1) contained C_5_–C_60_ hydrocarbons with functional groups including alkanes, alkenes, cycloalkanes, alcohols, esters, aromatics, and nitriles. Gasoline-range compounds (C_4_–C_12_), especially aromatics, nitriles, and alcohols, peaked at 700 °C (40.62%). Diesel-range compounds dominated at 600 °C (46.73%) and 800 °C (58.03%), mostly alcohols and cycloalkanes. Alcohols increased with temperature, while alkanes and esters declined. Motor oil-range (C_21_–C_35_) compounds included long-chain alkanes, esters, and PFAS-derived fluoroesters (e.g., tetratriacontyl pentafluoropropionate) used as additives in the manufacturing process of the FMs [30]. C_20_+ hydrocarbons decreased with temperature, while lighter alcohols and halogenated alkanes increased. Heavy hydrocarbons (C_36_+, >500 g/mol) dropped from 19.18% at 500 °C to 10.18% at 800 °C, indicating thermal cracking to lighter fractions. In contrast, Sun et al. [31] reported 70–72% of the liquid product obtained from pyrolysis of face masks at 440 °C being C_36_+.

Motor oil and heavy hydrocarbon compounds decreased with increasing temperature, with the most dramatic decrease occurring between 600 (29.56%) and 700 °C (18.64%), primarily due to the reduction in alkanes and esters between these temperatures. Branched alcohols, such as 2-octyl-1-Dodecanol and 2-hexyl-1-Dodecanol, made up between 15 and 40% of the total product, and were assumed to be produced from random scission of the polymer chains, producing primary alkyl radicals, followed by beta scission and intramolecular hydrogen transfer to produce secondary radicals and volatile 1-alkenes. The process of random scission of the polymer chain is referred to as back-biting and can result in the formation of different carbon-number compounds determined by the position at which the chain is broken [32]. Alcohols were produced due to oxygen in the nitrile glove. Furthermore, esters, such as phthalates used as plasticisers, could be reduced to alcohols, via an aldehyde intermediate [27]. Aromatic compounds constituted approximately < 6% of the condensable product up to 600 °C, but did rise with increasing temperature (8.17% at 800 °C), and included BTX compounds (styrene and benzene-related compounds); polycyclic aromatic hydrocarbons (PAHs), including naphthalene and its derivatives, such as naphthalene, 1,2-dihydro-1,1,6-trimethyl-; and N-containing aromatic compounds (NACs), including benzonitrile and aniline. Cycloalkanes were also a major functional group present in the condensable product, ranging from around 13.1% at 500 °C to 17.9% at 600 °C, which derived from face masks [33,34]. These compounds are due to cyclisation reactions occurring between two secondary radicals, which is known to be promoted by higher temperatures, as described by Zhang et al. [35].

**Figure 4:**
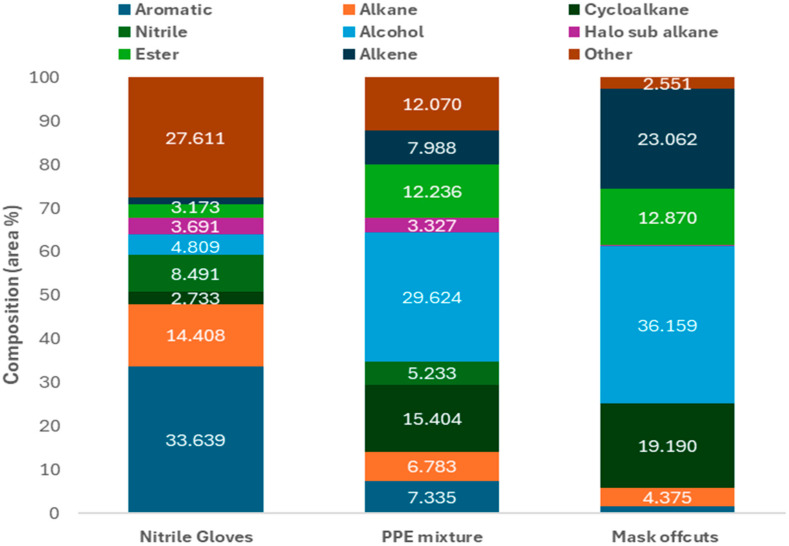
Comparison of condensable product obtained at 700 °C.

Many of the aromatics and larger alkanes were found to be thermal degradation products from pyrolysis of nitrile gloves, including Bis(2-hydroxy-5-methyl-3-[1-methylcyclohexyl]phenyl)methane, 1,4-benzenediamine, N4-(diphenylmethylene) N1,N1-diethyl-, and tetratetracontane. GC-MS of the condensable product from pyrolysis of nitrile gloves at 700 °C showed that the two most abundant functional groups were aromatics (33.64%) and alkanes (14.41%). Comparing the composition of the condensable products from the 50:50 mixture to that of the parent materials (Figure 4), it is suggested that the aromatic compounds usually found in NG pyrolytic oil are being broken down into cycloalkanes and smaller hydrocarbons, which can then form esters and alcohols due to the significant concentration of oxygen present from the pyrolysis decomposition products of the NGs.

#### 2.2.1. Gas Composition

**Figure 5:**
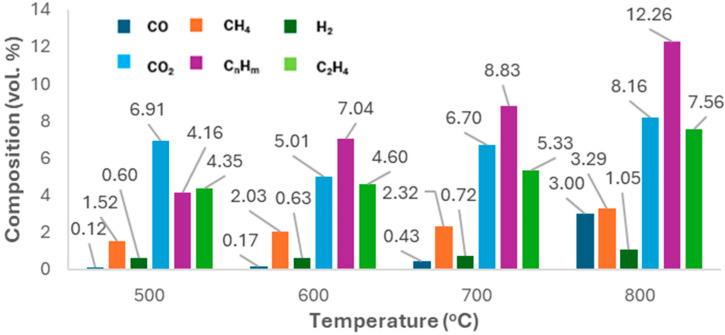
Composition of gas produced at different pyrolysis temperatures.

Figure 5 shows the compound distribution in pyrolysis gas, normalised by yield at each temperature. H_2_ dominated (30–35 vol.%), followed by C_2_–C_4_ (15–25 vol.%), ethylene (15–20 vol.%), and methane (10–15 vol.%). CO was minor (1–6 vol.%), while CO_2_ was more prominent (10–20 vol.%). Wang et al. [36] found that pyrolysing face masks from 550–650 °C increased H_2_ from 15.8 to 31.4 vol.%, while C_2_ and C_3_ gases fell, and methane rose from 18.2 to 26.4 vol.%, indicating further dehydrogenation of light hydrocarbons. They also saw a drop in C_1_–C_4_ gases from 84 to 69 vol.%. In this study, C_1_–C_4_ gases ranged from 45 to 55 vol.% at 600–800 °C, following Wang’s trend. COₓ and C_4_ gases also declined with temperature.

#### 2.2.2. Scale-Up Auger Reactor Test

**Figure 6:**
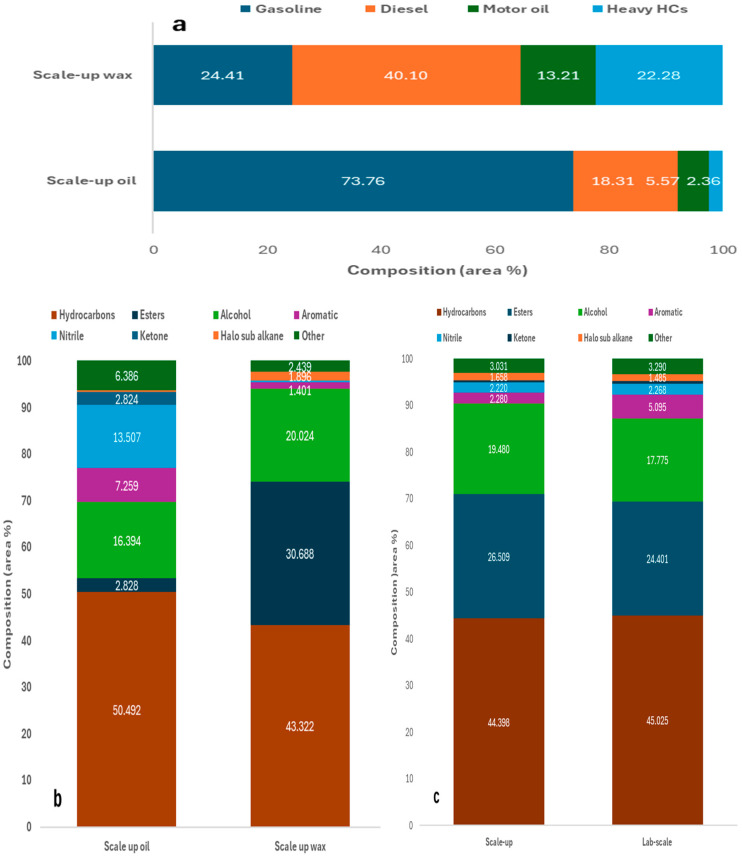
(**a**) Carbon number distribution of scale-up oil and wax, and functional groups present in (**b**) scale-up oil and wax, and (**c**) combined scale-up (85 wt.% wax, 15 wt.% oil) vs lab-scale condensable product at 500 °C.

The condensable product yield from pyrolysis of the PPE mixture at 500 °C was calculated as 63.0 wt.%, giving a gas yield of 27.4 wt.%. There was a high proportion of wax produced, indicating the need to adjust the residence time to obtain maximum oil yield. These yields correlated well with the obtained experimental yields of 67.6 wt.% for condensable product and 22.7 wt.% obtained for gas yield at the lab scale. The melting point of the wax produced was estimated to be between 220 and 280 °C through heating to 300 °C and cooling the sample while checking periodically to determine when the oil solidified into wax. The carbon number distribution and most abundant functional groups present for the scale-up oil and wax, and how their composition compares with the lab-scale condensable product obtained, is illustrated in Figure 6.

The carbon number distribution and concentration of functional groups present in the condensable product derived from the lab-scale experiments was found to be intermediate to those of the oil and wax produced from the auger reactor test, at the same temperature. This was validation that the composition of the product obtained at the lab scale could be replicated at a significantly higher scale. Furthermore, the scale-up condensable product was estimated to be 85 wt.% wax and 15 wt.% oil, shown by the close correlation between the lab-scale results and carbon number distribution and functional groups expected for this mixture when calculated from the scale-up oil and wax results. The notable increase in aromatics present in the lab-scale condensable product was thought to be due to the longer residence time used, which was corroborated by the higher wax proportion found in the scale-up product, as wax formation is favoured by lower residence times.

### 2.3. Analysis of Contaminants Present in PPE Oil and Associated Challenges for Oil Refinery Entry

Table 2 below compares the contamination levels found in the PPE pyrolysis oil to known contaminant levels for industrial crackers. As seen, the oxygen concentration of the oil greatly exceeds the threshold value for entry to industrial crackers. Oxygen is primarily derived from the nitrile gloves. Sulphur concentration was found to be comfortably within the limit and may be beneficial at this low concentration by restricting the formation of CO and CO_2_ through binding to the catalyst Ni active sites and preventing steam reforming [20]. Nitrogen values were found to be well above the allowed concentration. Nitrogen is known to cause catalyst deactivation in a range of refinery units, including hydrocrackers; cause formation of explosive gum within steam crackers; and lead to the formation of ammonia and nitriles, which can cause the product obtained to be out with specifications [20]. The metals Ca, Na, and Fe were all found to be slightly above the lower limit, but this could be simply remedied by mixing with naphtha to dilute the concentration before use in the steam cracker. Organic and inorganic chlorides facilitate corrosion of equipment, such as metal surfaces, heat exchangers, and downstream facilities [20]. Although Br is not greatly out of specification, Cl and F both significantly exceed the threshold values, indicating that the PPE oil would require pre-treatment, through dechlorination and fluorine removal, to reduce the concentration of these halogens as well as reduce N_2_ levels, before entry to the steam cracker. Recently, Jeon et al. [37] showed that ZSM-5 catalysts can remove most Cl from organochlorine compounds at 180 °C, while KOH solution can eliminate half of the Cl from inorganic chlorine compounds below 40 °C. Although the water content, density, and viscosity of the oils were not analysed, these were thought to be within specification, due to the appearance of the oil, with the oil being pourable and floating on water, indicating that the viscosity and density were both like that of water. The water content of the oil was also assumed to be within specification, as most of the water could be separated in a vial through simple gravity separation. To confirm, these should be measured by the relevant ASTM standards, as well as the remaining unanalysed properties.

### 2.4. Kinetic Analysis

#### 2.4.1. Model-Free Methods

**Figure 7:**
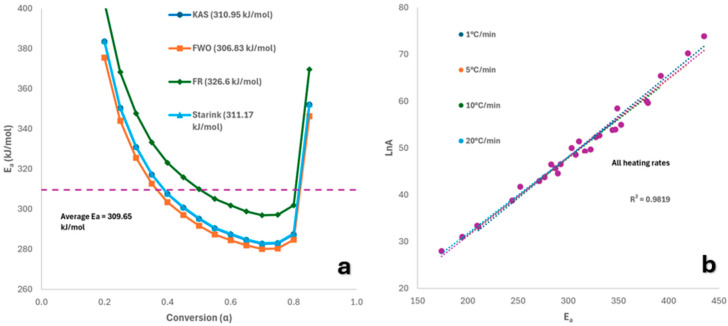
Plots of (**a**) Conversion vs E_a_ for model-free methods, and (**b**) E_a_ vs lnA for all reaction mechanisms.

Table 3 shows E_a_ and lnA values across 0.2–0.85 conversion, with this range chosen because, at lower values, the R^2^ value calculated was less than 0.95, and the activation energy calculated was significantly higher than the average displayed for each model in Table 3. This indicates that the initial reaction mechanism may differ from the one observed at conversions between 20 and 85% due to moisture release or the decomposition of highly volatile components, which can distort kinetic parameter estimation for the primary decomposition [39]. Furthermore, from Table 1, the expected total mass lost during pyrolysis of the PPE mixture was approximately 89 wt.%, meaning that higher degrees of conversion could not be considered. Including higher conversions would thus extend beyond the physical endpoint of the reaction, leading to inaccurate modelling. E_a_ trends were consistent across models, excluding FR. Average E_a_ values were KAS (310.95), FWO (306.83), FR (326.60), and Starink (311.17 kJ/mol), with standard deviations of ~30–32 kJ/mol. E_a_ varied notably with conversion (280.17–383.74 kJ/mol), suggesting a multi-step process, likely due to synergistic effects and complex mechanisms involving competing or secondary reactions. The overall average E_a_ (excluding FR) was 309.65 kJ/mol, higher than for typical waste plastics (267–298 kJ/mol) [40] or individual PPE (237 kJ/mol) [41] and nitrile gloves (224.36 kJ/mol) [42]. This difference may stem from the thermal range used or additives in the PPE. Higher E_a_ at low conversions likely reflects the energy needed to break nitrile rubber cross-links and initiate cyclisation. Furthermore, the higher activation energy calculated could be due to the improvement in thermal stability of the polyproyplene mask offuts through the addition of nitrile, as shown previously when these two materials are blended together [43]. At higher conversions, chain scission dominates, lowering energy requirements [33]. The kinetic compensation effect (KCE) is a phenomenon often observed in which there is direct proportionality between the activation energy and pre-exponential factor [44]. As seen from Figure 7, pyrolysis of mixed PPE waste appears to follow the KCE, denoting that changes in reaction conditions, such as changing the pyrolysis temperature, will lead to a predictable alteration in both E_a_ and A, signifying that they are not independent of one another. This knowledge is useful, as it would aid in the development of accurate kinetic models, reactor design, and optimisation, and in the understanding of the underlying reaction mechanism occuring during pyrolysis [45].

The values of E_a_ calculated using the KAS and Starink methods showed very good correlation, as seen in Figure 7, while the FR method returned notably higher values due to systematic errors related to its high sensitivity to experimental noise [46,47]. As can be seen in Figure S4, for all the kinetic models used, the slopes of the trendlines followed the same pattern of decreasing in gradient as conversion increased and returned reasonably similar values for each corresponding conversion between 0.2 and 0.8. This indicates that a single reaction mechanism can be used to describe the pyrolysis of FMs and NGs between 20 and 80% conversion. At conversions less than 20% and greater than 80%, there were noticeable variations in the slope of trendline, which can be explained due to the generation of volatiles initially, and the formation of radicals later in the process.

#### 2.4.2. Determination of Reaction Order by the CR Method

The Coats–Redfern (CR) method was used at four heating rates (1, 5, 10, 20 °C/min) to determine E_a_ and lnA for various reaction models. The CR method fits TG data to kinetic models to identify the most suitable mechanism, chosen based on both the highest R^2^ and E_a_ values closest to the average from model-free methods—since the highest R^2^ does not always mean the best fit. Due to fillers and melting behaviour, polymer mixtures degrade through complex, multi-step processes. However, the PPE mixture pyrolysis in N_2_ (20–85% conversion) was treated as single-step, as E_a_ remained reasonably constant. Table 4 lists E_a_, lnA, and R^2^ values for each reaction model (see Table S3), showing the main degradation stage between 400–550 °C (Figure 1c,d). All models gave high R^2^ (0.9960–0.9993); the best E_a_ match to model-free results (309.65 kJ/mol) came from D2 (310.51 kJ/mol, R^2^ = 0.9982) and D4 (325.71 kJ/mol, R^2^ = 0.9976), suggesting that diffusion plays a key role in the mechanism. However, E_a_ alone cannot confirm mechanism, so the Criado method was also applied for further validation.

**Figure 8:**
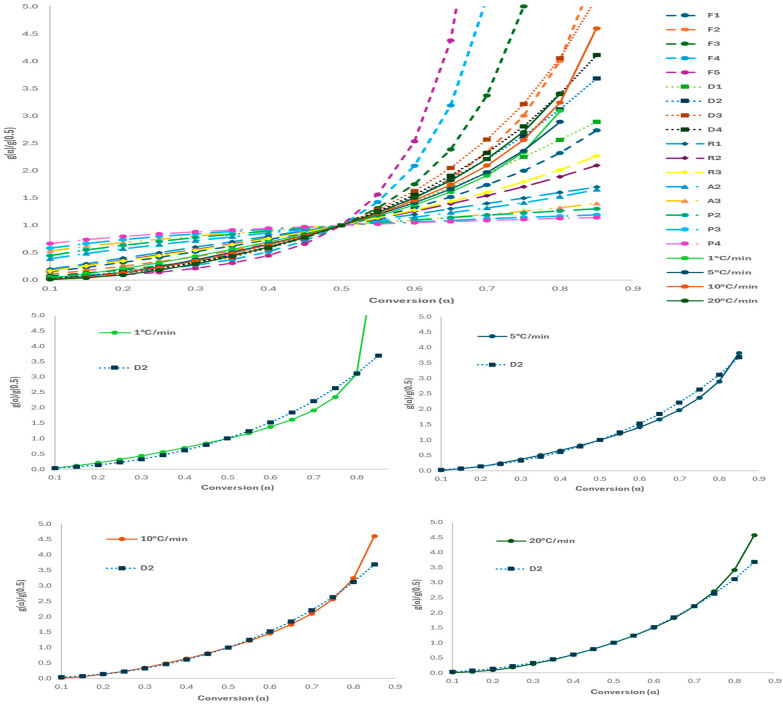
Comparison of theoretical and experimental master plots.

The Criado (or Master Plots) method allows identification of the reaction mechanism without determination of the activation energy and pre-exponential factor, by comparing theoretical master plots with graphs prepared using experimental data, and was previously applied to a range of processes, including plastic pyrolysis. This study entailed preparing theoretical master plots for the range of reaction models outlined in Table S3, along with experimental curves for each of the four different heating rates. Figure 8 outlines the experimental plots obtained using all heating rates, and shows how they compare to the master plots for each reaction mechanism. For conversions between 0.1 and 0.8, there is significant overlap between the theoretical plot for a D2 reaction model with the experimental curve for 10 °C/min. There was also reasonable correlation between the experimental curve and theoretical plots for F2 and D1, for α values between 0.1 to 0.7, and D4, throughout the entire range of conversions. However, the Ea values obtained using the CR method for these reaction models all differed significantly more than for the D2 model. Therefore, these models are less relevant for accurately outlining the thermal degradation of the PPE mixture. As a result, the 2-dimensional diffusion (or Valensi) kinetic model was determined to be the most relevant for pyrolysis of a mixture of FMs and NGs. Pyrolysis of face masks has been shown to follow a D1 model [33], whereas nitrile-butadiene gloves follow a D3 model [42], suggesting that the determined 2D model for the mixture is intermediate to the two parent materials.

When the full range of conversions were considered (0.1 to 0.85), the average activation energy calculated was 330.33 kJ/mol, due to the significantly higher activation energy calculated for 10 and 15% conversion. The closest correlation for the reaction order using the CR method was D3 (317.35 kJ/mol, R^2^ = 0.9985), shown in Table S4. Although F3 returned the next closest value (299.10 kJ/mol, R^2^ = 0.9988), there was little overlap between the F3 Criado plot and experimental plots at all heating rates. D2 and D4 provided the next closest match, suggesting that diffusion through a product layer is the rate-limiting step. This was corroborated using the Criado method, which illustrated a close match for all heating rates for both D2 and D3 reaction models (Figure S5).

## 3. Materials and Methods

### 3.1. Materials

**Figure 9:**
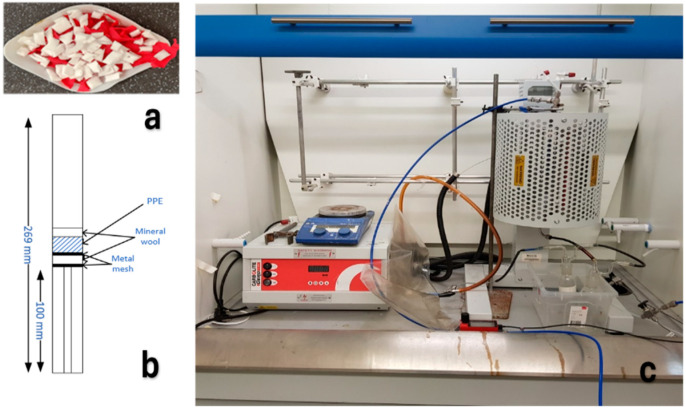
(**a**) PPE raw material, (**b**) Reactor configuration, and (**c**) Pyrolysis apparatus.

Alpha Solway Ltd. (Annan, Dumfriesshire, UK) provided surgical mask offcuts and powder-free disposable nitrile gloves (Sky98, Rhode, RI, USA). As they were unused and uncontaminated, there was no need to perform any drying or sterilisation. However, the PPE items were cut into small pieces, around 2–3 mm in size (Figure 9a). This size reduction step was conducted to enhance the total surface area available for heat transfer during pyrolysis, to ensure that uniform heating and thermal degradation of the material occurred. A 50:50 mass mixture of FM:NG was chosen for the pyrolysis experiments, as they are representative of the typical composition of PPE waste from the sector.

### 3.2. Pyrolysis Experimental Set-Up and Method

The experimental set-up used for pyrolysis (illustrated in Figure 9b,c) was carefully designed to allow for maximal conversion of PPE to condensable product. The pyrolysis reactor itself was a stainless-steel (T316) fixed-bed reactor chosen due to its simplicity, reliability, and practicality [48].

The reactor has dimensions of 269 mm, 16 mm, and 2 mm for length, inner diameter, and wall thickness. The PPE feed (1.5 g total mass) was elevated using a small metal pipe to 100 mm from the bottom of the reactor, and held in place with metal mesh, between layers of mineral wool for insulation (Figure 9b). This configuration, which ensured that the sample was situated at the centre of the furnace (EVA 12/600B Vertical Tube Furnace, Carbolite, Hope, UK), resulted in a total volume of 216 cm^3^. The thickness of the PPE layer was approximately 15 mm. The maximum temperature was set using the heating controller, with preliminary tests proving that below 500 °C, waxing was considerable, causing blockages in the reactor outlet and making it challenging to differentiate between the feed that was not volatilised, char, and wax. For this reason, the temperature was varied between 500 and 800 °C. The condensable product yields and overall conversion achievable for thermal pyrolysis of the PPE feed were then obtained. As part of the apparatus, the pyrolysis vapour exiting the reactor passed through a series of condensing bottles, with the first at ambient temperature (approximately 15 °C), and the second cooled to below 0 °C in a salt–ice bath. It was within these bottles that the reaction vapours were cooled sufficiently to condense into both liquid oil and solid wax, with most of the product from all tests collected at ambient temperature (high-temperature condensate, HTC). Product distribution for the low-temperature condensate (LTC) was below 15 wt.% of the total condensable product yield for all tests and appeared as a light-yellow liquid oil. The entire reaction was conducted at standard atmospheric pressure, using a gas flow of 100 mL/min N_2_ to ensure the inert conditions required for pyrolysis. At this flowrate, the vapour residence time was calculated as 8.41 s, which classified the process as intermediate pyrolysis [16]. The pyrolysis process yielded three main outputs, with the product distribution calculated at each temperature evaluated. The following equations were used to calculate the overall conversion (α), and condensable product, solid residue, and gas yields, respectively, in wt.%.

(1) $$\alpha \left(wt.\%\right)=\left(\frac{m_{0}-m_{f}}{m_{0}}\right)\;\times 100$$

(2) $$Condensable\;Product\;yield\left(wt.\%\right)=\left(\frac{∆m\;of\;condensing\;bottles}{m_{0}}\right)\times 100$$

(3) $$Solid\;Residue\;yield\left(wt.\%\right)=100-\alpha$$

(4) $$Gas\;yield\left(wt.\%\right)=\alpha -Condensable\;Product\;yield$$

where m_o_ and m_f_ are the initial and final sample mass, and ∆m is the change in mass.

The pyrolysis reaction was conducted using a controlled heating rate, which was set to the maximum possible (100 °C/min) due to the known capability of fast pyrolysis to prioritise liquid oil yield [16], and to ensure total thermal breakdown of the PPE waste while attempting to minimise undesirable by-products through secondary reactions. This heating rate was used for all tests performed, to ensure that the only impact on the product yields and distribution was due to the varying pyrolysis temperature. The furnace was programmed using the heating controller to increase the temperature of the reactor from ambient to the set value as rapidly as possible, to ensure uniform heating of the full sample, to prevent thermal shock from causing anomalous results. The heating rate was kept constant to ensure that the results obtained were reproducible, as it is one of the key variables in determining the carbon number distribution of the products, as well as the overall conversion [49].

### 3.3. Materials Characterisation

Physicochemical characterisation of the PPE mixture, and solid and liquid products from pyrolysis, included proximate and ultimate analysis, HHV estimation, and FTIR. The proximate analysis was determined using TGA (Linseis STA PT 1600, New Delhi, India). The moisture content was determined by firstly heating 20 mg of the PPE mixture to 110 °C in 50 mL/min of N_2_ gas and maintaining this temperature for 15 min. Next, the sample was ramped up to 900 °C at 20 °C/min and held for 40 min. Lastly, the sample was cooled to 750 °C, held for 40 min, and the gas switched to air, to allow combustion to occur and determine the ash content. Fixed carbon (FC) was estimated by the difference (100-MC-VM-FC). Ultimate analysis was conducted using an Exeter CE-440 Elemental Analyser (North Chelmsford, MA, USA), which measured the carbon (C), hydrogen (H), and nitrogen (N) content of the sample, with the remainder assumed to be oxygen (100-C-H-N). Approximately 20–25 mg of the sample was weighed and added to a crucible and analysed between ambient temperature and 900 °C in an inert N_2_ atmosphere. To determine the minimum N_2_ flowrate required to ensure that external mass transfer limitations did not impact the overall reaction rate, a series of tests using a 20 °C/min heating rate and a range of gas flowrates were carried out. As seen in Figure S6, at 50 mL/min, the overall mass loss of the PPE mixture samples was observed to plateau, indicating that the reaction rate was maximal, and therefore, the TGA tests were conducted using 50 mL/min of N_2_. For proximate analysis, the heating rate was kept constant at 10 °C/min. The kinetic parameters of PPE pyrolysis were investigated using the same TGA apparatus and conditions; however, a range of different heating rates were used (1, 5, 10, and 20 °C/min). Values between 1 and 20 °C/min were chosen, as at higher heating rates (30 °C/min), the results were deemed to be inaccurate since the expected trend of rising conversion temperatures was not observed. This could be due to the generation of a temperature gradient as a result of heat transfer limitations, where the temperature on the surface of the plastic differed from the core temperature of the sample, leading to anomalies in the weight loss measurement [50]. All tests were repeated to ensure reproducibility. The dry and ash-free (daf) calorific value of both PPE parent materials was estimated using the following equation [51]:

HHV (daf, MJ/kg) = 0.3491C + 1.1783H + 0.1005S − 0.1034O − 0.015N − 0.0211A(5)

FTIR spectroscopy of both FMs and NGs was conducted to determine the presence of various functional groups using a Thermo Fisher Nicolet IS5 (Thermo Fisher Scientific, Waltham, MA, USA) with an ID5 ATR accessory, with spectra collected and analysed between 4000 and 550 cm^−1^. Identification of the condensable compounds was achieved by referring to mass spectral libraries. GC-MS analysis performed on the condensable product from PPE waste pyrolysis provided an estimation of the relative abundancy of different compounds in the mixture, which was used to compare the relative distribution of functional group and carbon number ranges within the product mixture. Several studies have reported product compositions using similar semi-quantitative GC-MS approaches [52,53,54,55]. Oil/wax samples were run on a Shimadzu 2010SE instrument, with a Restek 5 HTI column (30 m × 0.25 × 0.25) (Restek, Bellefonte, PA, USA) using acetone as the solvent. The sample was injected at 290 °C, at a pressure of 37.1 kPa, and a total flow of 34.2 mL/min. The oven was ramped from 50 °C to 310 °C at heating rates varying from 4 to 10 °C/min, and held at 90, 120, and 300 °C for several minutes. ICP-MS was used to determine the concentration of a range of different metal and non-metal ions and performed on an Agilent 7900 single quad ICP-MS (Santa Clara, CA, USA). The gases produced were analysed using a Cubic–Ruiyi Gasboard 3100 gas analyser that provided the relative quantities of six gas compounds present, including hydrogen (H_2_), methane (CH_4_), ethylene (C_2_H_4_), carbon dioxide (CO_2_), carbon monoxide (CO), and larger hydrocarbon gases (C_3+_).

### 3.4. Auger Reactor Scale-Up Test

To determine the reproducibility of the product yields and compositions obtained at the lab scale, fixed-bed experiments when the process was scaled-up, a series of preliminary feeding tests at one pyrolysis temperature of 500 °C was performed, in duplicate, using a custom-made auger reactor of length 61 cm and diameter of 6 cm (pictured in Figure S7a–c). The furnace used was electrical, and purchased from Zhengzhou Protech Technology Co., Ltd. (Zhengzhou, China). The feeding rate of the PPE mixture (50:50 mass ratio of FMs and NGs), was kept at the lowest value to minimise operational issues due to blockages. When measured, this feeding rate was determined to be 0.36 kg/h (240 times the 1.5 g used for lab-scale tests). The reactor configuration consisted of a feed hopper, screw conveyor, char pot, and two sequential Graham condensers, maintained at ambient temperature (15 °C) [56]. Nitrogen was passed through the system to ensure an inert atmosphere, with oil and wax samples collected separately, and the gas produced vented to the atmosphere via an extraction system. Heating tape set at 250 °C was wrapped around both the reactor outlet and condensers to try to minimise wax formation. By weighing the mass of oil and wax obtained, assuming a char yield equal to the value 9.73 wt.% produced in the lab-scale tests, since it was not possible to separate the char and wax produced, and determining the gas yield via the difference, the overall product distribution was calculated. The oil and wax samples were analysed by GC-MS, with the carbon number distribution and abundance of functional groups compared with the condensable product obtained at the lab scale. The melting point of the wax was estimated by inserting a small sample into an oven at elevated temperature to convert it to liquid, then cooling the oven and determining the temperature at which the sample became solid wax.

### 3.5. Kinetic Triplet Determination

The kinetic triplet of pyrolysis of the FM/NG mixture (activation energy, pre-exponential factor, and reaction order) was determined using the method described below. Determination of the kinetic parameters and reaction mechanism of waste PPE pyrolysis is vital to enable optimal reactor design and identification of desired operating conditions, allowing effective scale-up of the pyrolysis process [39,57,58]. The thermal decomposition of a solid fuel can be generalised as:

(6) $$Solid\;fuel\;\overset{k}{\to }Volatile\;matter\;+\;char$$

where k is the reaction rate constant, dependant on temperature (T), and can be calculated using the Arrhenius equation:

(7) $$k\left(T\right)=Aexp\left(\frac{-E_{a}}{RT}\right)$$

where E_a_ is the activation energy (kJ/mol), R is the universal gas constant (8.314 × 10^−3^ kJ/mol K), and A is the pre-exponential factor (usually reported in s^−1^).

The rate at which a solid fuel is converted into products can be expressed as:

(8) $$\frac{d\alpha }{dt}=k\left(T\right)f\left(\alpha \right)$$

where α is the overall conversion on a mass basis, t is time, k(T) is equal to the temperature-dependant rate constant, and f(α) is the reaction mechanism rate expression. The degree of conversion can be determined using:

(9) $$\alpha =\frac{m_{i}-m}{m_{i}-m_{f}}\;$$

where m_i_, m, and m_f_ are the initial mass, mass at any reference time, and final mass, respectively.

By substituting Equation (7) into Equation (8), the following expression is obtained.

(10) $$\frac{d\alpha }{dt}=k\left(T\right)f\left(\alpha \right)=Aexp\left(\frac{-E_{a}}{RT}\right)f\left(\alpha \right)$$

The heating rate, β (°C/min), can be expressed as:

(11) $$\beta =\frac{dT}{dt}=\frac{dT}{d\alpha }x\frac{d\alpha }{dt}$$

By introducing the above expression for heating rate into Equation (10), a new expression for $\frac{d\alpha }{dt}$ is developed.

(12) $$\frac{d\alpha }{dt}=\beta \frac{d\alpha }{dT}=Aexp\left(\frac{-E_{a}}{RT}\right)f\left(\alpha \right)$$

Integration of Equation (12), assuming E_a_, A, and β are constants between α = 0 at T = 0 and the temperature at each conversion as T, the following expression is obtained.

(13) $$\int_{0}^{\alpha }\frac{d\alpha }{f(\alpha )}=g\left(\alpha \right)=\frac{A}{\beta }\int_{0}^{T}\exp\left(\frac{-E_{a}}{RT}\right)dT$$

with *g*(*α*) being the integral form of the reaction mechanism, which can be found in Table S3. Therefore, if u can be defined by the equation below, a general expression for g(α) can be formed:

(14) $$u=\frac{E_{a}}{RT}$$

(15) $$g(\alpha )=\frac{AE_{a}}{\beta R}\int_{u}^{\infty }\frac{exp(-u)}{u^{2}}du=\frac{AE_{a}}{\beta R}p(u)$$

This general expression can be used to determine the kinetic triplet for pyrolysis. To reduce the complexity of the calculations, an approximation for *p*(*u*) is used, as outlined in Section 3.6 and Section 3.7.

### 3.6. Model-Free Kinetic Methods

Isoconversional (or model-free) methods of determining the activation energy for the thermal decomposition of solid fuels have the advantage of not requiring any prior knowledge of the reaction mechanism. In this study, the kinetic parameters of the thermal degradation of the PPE mixture were determined using four different model-free methods that have been used extensively, including the KAS, FWO, Friedman, and Starink methods. To calculate the activation energy, each of these methods uses different equations, as outlined below.

#### 3.6.1. Kissinger–Akahira–Sunose (KAS)

The KAS method makes use of the assumption that E_a_ and A are independent of both the temperature and conversion to calculate the kinetic parameters. Using the approximation below for *p*(*u*) given by Equation (16), and substituting this expression into Equation (15), and then taking the natural logarithm, the KAS method can be expressed by Equation (17).

(16) $$p\left(u\right)=T^{2}\exp\left(\frac{{-E}_{a}}{RT}\right)$$

(17) $$\ln\left(\frac{\beta }{T^{2}}\right)=\ln\left(\frac{AR}{E_{a}g(\alpha )}\right)-\frac{E_{a}}{RT}$$

Plotting $\ln\left(\frac{\beta }{T^{2}}\right)$ against 1/*T* should give a straight line with gradient equal to $\frac{-E_{a}}{R}$, from which the activation energy can be calculated.

#### 3.6.2. Flynn–Wall–Ozawa (FWO)

The FWO method again uses an approximation for *p*(u), as seen in Equation (18) below, which, when substituted into Equation (12), gives the following expression.

(18) $$p\left(u\right)=\exp\left(-1.052\frac{E_{a}}{RT}-5.0331\right)$$

(19) $$\ln\left(\beta \right)=\ln\left(\frac{AE_{a}}{Rg(\alpha )}\right)-5.331-1.052\frac{E_{a}}{RT}$$

Similarly to the KAS method, a plot of $\ln\left(\beta \right)$ against *1/T* should give a straight line with gradient equal to $\frac{-E_{a}}{R}$, from which the activation energy can be calculated.

#### 3.6.3. Friedman (FR)

Unlike the KAS and FWO methods, which use the integral forms of the reaction mechanism, the FR method uses the differential part and is obtained by taking the natural logarithm of Equation (12).

(20) $$\ln\left(\frac{d\alpha }{dt}\right)=\ln\left(\beta \frac{d\alpha }{dT}\right)=\ln\left(Af\left(\alpha \right)\right)-\frac{E_{a}}{RT}$$

By assuming that conversion is only dependent on the rate at which mass is lost, the activation energy can be calculated over a wider range of conversions. A plot of $\ln\left(\beta \frac{d\alpha }{dt}\right)$ against 1/T should give a straight line with gradient equal to $\frac{-E_{a}}{R}$, from which the activation energy and pre-exponential factor can be calculated.

#### 3.6.4. Starink

The equation for calculating the kinetic parameters using the Starink method is shown below.

(21) $$\ln\left(\frac{\beta }{T^{1.92}}\right)=Const-1.0008\frac{E}{RT}$$

A plot of $\ln\left(\frac{\beta }{T^{1.92}}\right)$ against 1/T results in a slope with gradient equal to $\frac{-1.0008E_{a}}{T}$, which can be used to calculate the activation energy.

### 3.7. Model-Fitting Methods

Model-fitting methods for calculating the kinetic parameters of a reaction use specific mathematical models to determine the reaction mechanism by evaluating which model accurately fits the TGA data generated.

#### 3.7.1. Criado (Master Plots)

To determine the reaction mechanism for the thermal decomposition of the PPE mixture, master plots (MPs) were created using the integral form of Equation (10), which is:

(22) $$g\left(\alpha \right)=\frac{AE}{\beta R}P(u)$$

where u is equal to $\frac{E}{RT}$, and P(u) is the integral:

(23) $$P\left(u\right)=\int_{\infty }^{u}-\left(\frac{e^{-u}}{u^{2}}\right)du$$

Although P(u) does not have a definitive solution, Doyle’s approximation [59] can be used to give results that are sufficiently accurate.

(24) $$P\left(u\right)=0.00484exp\;(-1.0516u)$$

The Criado method assumes that the decomposition process is a single step, where A and E_a_ are treated as constants, and g(0.5) is taken as a reference point, and can be calculated using Equation (25).

(25) $$g\left(0.5\right)=\frac{AE}{\beta R}P(u_{0.5})$$

By combining Equations (17) and (20), the following ratio is obtained:

(26) $$\frac{g(x)}{g(0.5)}=\frac{P(u)}{P(u_{0.5})}$$

The most common reaction mechanisms for the thermal degradation of solid-state fuels are given in Table S3. By plotting the theoretical g(x)/g(0.5) against conversion, and experimental P(u)/P (u_0.5_) vs. conversion, the most suitable reaction mechanism was determined by matching the theoretical values to the experimental results at different conversions.

#### 3.7.2. Coats–Redfern (CR)

The CR method utilises the integral form of the reaction mechanism and has been commonly used to determine the kinetic parameters for thermal degradation of solids. Equation (28) below is derived through expansion and simplification of Equation (15), with an appropriate expression for g(α) selected from Table S3.

(27) $$\ln\left(\frac{g(\alpha )}{T^{2}}\right)=\ln\left(\frac{AR}{\beta E_{a}}\right)-\frac{E_{a}}{RT}$$

To determine the activation energy and frequency factor, $\ln\left(\frac{g\left(\alpha \right)}{T^{2}}\right)$ is plotted against 1/T for a range of temperatures, corresponding to different values of α. The reaction model is then determined through comparison of the activation energy calculated using the CR method to the average value obtained using the model-free methods, with the model resulting in the closest match chosen to describe the reaction mechanism.

### 3.8. Determination of the Pre-Exponential Factor

After determining the most suitable reaction mechanism identified using the method described in Section 3.7, the pre-exponential factor was calculated using the expressions for the intercept of the plots for each kinetic model.

(28) $$\mathrm{KAS}=\ln\left(\frac{AR}{E_{a}g(\alpha )}\right)$$

(29) $$\mathrm{FWO}=\;\ln\left(\frac{AE_{a}}{Rg(\alpha )}\right)-5.331$$

(30) $$\mathrm{FR}=\ln\left(Af\left(\alpha \right)\right)$$

## 4. Conclusions

Treatment of mixed PPE waste by pyrolysis is demonstrated as a potential solution for management. This study found that at temperatures up to 600 °C, around 77 wt.% of condensable oil and wax can be recovered, with less than 10 wt.% solid residues. GC-MS analysis showed that the wax (17 wt.%, C_36_+) was rich in alcohols (26.11%), esters (20.49%), cycloalkanes (17.89%), alkenes (9.95%), and alkanes (9.44%). Diesel-range compounds dominated (46.73%) due to high alcohol and cycloalkane content. At 700 °C, wax dropped to 11 wt.% as gasoline-range compounds, including aromatics (9.85%) and nitriles (5.23%), increased. The gas produced at all temperatures contained CO, CO_2_, H_2_ (30–35 vol.%), CH_4_, C_2_H_4_, and larger hydrocarbons (45–55 vol.%). Auger reactor experiments confirmed high condensable yields, with combined product composition falling between lab-scale oil and wax samples. The pyrolysis oil exceeded limits for oxygen, nitrogen, and halogens, indicating that hydrotreatment and dehalogenation are needed if the oils are to be upcycled in a steam cracker. Metal content was near limits but could be lowered by blending with naphtha. The kinetic analysis resulted in an average E_a_ of 309.65 kJ/mol and an A of 2.5 × 10^25^ s^−1^, while the CR and Criado analysis indicated a 2D diffusion model, which provides essential information for reactor scale-up. Future research should explore catalyst-assisted pyrolysis to reduce contaminants in the produced oils and assess the techno-economic feasibility of the process.

## Figures and Tables

**Table 1:** Proximate and ultimate analyses of the mask offcuts and NGs.

**Proximate Analysis (wt.%)**	**Mask Offcuts**	**Nitrile Gloves**	**Mixture**
Moisture (MC)	0.72	0.93	0.05
Volatile Matter (VM)	97.52	84.74	88.98
Fixed Carbon (FC)	1.35	3.72	2.03
Solid Residue (SC)	0.41	10.62	8.95
Total	100.00	100.00	100.00
**Ultimate Analysis (wt.%)**	**Mask Offcuts**	**Nitrile Gloves**	**Mixture**
C	85.45	73.20	79.33
H	14.45	8.58	11.52
O	0.00	11.61	5.81
N	0.10	6.62	3.36
Total	100.00	100.00	100.00
Higher heating value, daf (MJ kg^−1^)	46.86	34.36	40.61

**Table 2:** Comparison of contamination levels in PPE pyrolysis oil and known contaminant levels for industrial crackers.

Property (Units)	Limit	PPE Mixture	Reference
Total Oxygen (ppm)	>100	29,200 (est. from GC-MS)	[20]
S (wt.%)	>1.5	<0.10 (est. from GC-MS)	[38]
5% pt (°C)	>510	200.18	[38]
95% pt (°C)	>720	467.90	[38]
FBP (°C)	>700	517.56	[38]
N total (wt ppm)	>100	38,700	[20]
Ca (ppm)	>0.5	2.00	[20]
Na (ppm)	>1	3.41	[20]
Ni (ppm)	>100	0.22	[20]
Fe (ppm)	>2	2.71	[38]
Cl (ppm)	>3	800 (est. from GC-MS)	[20]
Br (ppm)	>20	0.28	[38]
F (ppm)	>2	15,600 (est. from GC-MS)	[20]
Dioxines (ppb)	Detectable	Not detectable	
Furanes (ppb)	Detectable	Not detectable	

**Table 3:** Activation energy and pre-exponential factors of PPE pyrolysis using model-free method.

Conversion	KAS		FWO		FR		Starink	
α	E_a_ (kJ/mol)	lnA	E_a_ (kJ/mol)	lnA	E_a_ (kJ/mol)	lnA	E_a_ (kJ/mol)	lnA
0.1	530.80	90.11	515.08	88.34	559.04	87.96	530.81	82.94
0.15	428.12	70.50	417.69	69.20	449.93	67.33	428.23	63.56
0.20	383.59	62.20	375.49	61.14	403.05	58.69	383.74	55.38
0.25	350.44	56.21	344.08	55.34	368.23	52.49	350.62	49.48
0.30	330.88	52.73	325.57	52.00	347.65	48.88	331.08	46.08
0.35	317.23	50.36	312.66	49.72	333.27	46.42	317.45	43.76
0.40	307.54	48.71	303.50	48.14	323.06	44.72	307.77	42.15
0.45	300.70	47.57	297.04	47.05	315.85	43.54	300.93	41.05
0.50	295.12	46.66	291.78	46.18	309.97	42.61	295.36	40.17
0.55	290.46	45.90	287.39	45.47	305.08	41.84	290.70	39.46
0.60	287.42	45.43	284.54	45.02	301.85	41.36	287.67	39.01
0.65	284.60	44.98	281.90	44.60	298.89	40.92	284.85	38.60
0.70	282.75	44.69	280.17	44.32	296.96	40.64	283.01	38.34
0.75	282.95	44.72	280.40	44.35	297.14	40.69	283.20	38.40
0.80	287.52	45.44	284.81	45.05	301.91	41.46	287.78	39.15
0.85	352.04	55.90	346.27	55.14	369.54	52.27	352.25	49.46
Average (0.2–0.85)	310.95	49.39	306.83	48.82	326.60	45.47	311.17	42.89
Average (0.1–0.85)	332.01	53.26	326.77	52.57	348.84	49.49	332.22	46.69

**Table 4:** Values of E_a_ and lnA obtained using the CR method for different reaction models.

Model	1 °C/min		5 °C/min		10 °C/min		20 °C/min		Average		R^2^
	E_a_ (kJ/mol)	lnA	E_a_ (kJ/mol)	lnA	E_a_ (kJ/mol)	lnA	E_a_ (kJ/mol)	lnA	E_a_ (kJ/mol)	lnA	
F1	210.50	33.2	209.45	33.4	194.49	31.0	173.80	28.0	197.06	31.40	0.9968
F2	310.75	51.4	303.31	50.0	282.88	46.5	252.17	41.7	287.28	47.40	0.9987
F3	435.18	73.9	419.02	70.2	391.94	65.4	348.72	58.4	398.72	66.99	0.9960
D1	287.25	45.7	292.46	46.5	271.07	42.9	244.06	38.8	273.71	43.47	0.9993
D2	327.33	52.3	331.05	52.7	307.28	48.6	276.36	43.7	310.51	49.32	0.9982
D3	378.05	60.0	379.23	59.6	352.56	55.0	316.64	49.3	356.62	55.96	0.9963
D4	344.04	53.8	346.94	54.0	322.21	49.7	289.65	44.5	325.71	50.50	0.9976

## Data Availability

The authors will make the material available upon request.

## Supplementary Materials

The following supporting information can be downloaded at: https://www.mdpi.com/article/10.3390/molecules30163351/s1, Figure S1: GC-MS Chromatograms for PPE Mixture at 500, 600, 700, and 800 °C; Figure S2: GC-MS Chromatograms for Mask Offcuts and Nitrile Gloves at 700 °C; Figure S3: GC-MS Chromatograms for PPE Mixture from Auger reactor at 500 °C; Figure S4: Linear plots for determination of Ea using a. KAS, b. FWO, c. FR, and d. Starink kinetic methods; Figure S5: Comparison of theoretical and experimental master plots for full conversion range; Figure S6: N_2_ flowrate comparison using 20 °C/min heating rate; Figure S7: a. Hopper and feeding system, b. Auger reactor and furnace, and c. Condensers and sample bottle; Table S1: Composition (GC-MS, Area %) of the condensable product obtained through thermal pyrolysis of the PPE mixture at 500, 600, 700, and 800 °C.; Table S2: Composition (GC-MS, Area %) of the condensable product obtained through thermal pyrolysis of the Mask offcuts; Table S3: Common Reaction Mechanisms and their Differential and Integral Rate expressions and Nitrile gloves, and PPE mixture using the scale-up Auger reactor; Table S4: Reaction Mechanisms and their Differential and Integral Rate expressions for conversions between 0.1 and 0.85.
