# Supplementary material for: Pyrolysis of Polypropylene and Nitrile PPE Waste: Insights into Oil Composition, Kinetics, and Steam Cracker Integration
Source: Molecules. 2025 Aug 12;30(16):3351. doi: 10.3390/molecules30163351 (PMC12388647; doi:10.3390/molecules30163351)
Supplement: Supplementary file 1 [file molecules-30-03351-s001.zip › molecules-3810588-supplementary.pdf]

Supplementary Material

Table S1

Composition (GC-MS, Area %) of the condensable product obtained through thermal pyrolysis of the PPE mixture at 500, 600, 700, and 800°C

Condensable product at 500°C			
**C Number**	**Compound**	**MW**	**Area %**
C_5	D-Proline	115	0.726
C_6	Hexane, 1-chloro-	120	0.189
	2-Pentanone, 4-hydroxy-4-methyl-	116	0.294
	4-Pentenenitrile, 2-methylene-	93	0.204
	5-Cyano-1-pentene	95	0.125
	Aniline	93	0.171
C_7	6-Heptene-1-nitrile	109	0.495
	Heptanonitrile	111	0.329
	Cyclopentanecarbonitrile, 3-methylene-	107	0.172
	3-Hepten-2-ol, (E)-	114	0.735
	Cyclohexen-1-carbonitrile	107	0.225
	Silane, chloromethylphenyl-	156	0.173
C_8	5H-1-Pyrindine, 6,7-dihydro-	119	0.625
	Styrene	104	0.279
C_9	2,4-Dimethyl-1-heptene	126	1.585
	Nonane, 1-chloro-	162	0.366
	2-Methylindoline	133	0.182
	7-Methyl-1,6-octadiene	124	0.536
	Quinoline, 1,2,3,4-tetrahydro-	133	0.617
	1H-Indole, 5-methyl-	131	0.200
	Quinoline, 5,6,7,8-tetrahydro-	133	0.203
	1-Benzyl-1H-1,2,4-triazole	159	0.211
C_{10}	1H-Indene, 2,3-dihydro-4-methyl-	132	0.179
	(2,4,6-Trimethylcyclohexyl) methanol	156	1.356
	1,5,7-Octatrien-3-ol, 2,6-dimethyl-	152	0.245
	Cyclobutane, 1,3-diisopropenyl-, trans	136	0.256
	2,6-Octadien-1-ol, 2,7-dimethyl-	154	0.127
	Octane, 3,3-dimethyl-	142	0.312
	Naphthalene	128	0.175
	2-(1-Methylcyclopropyl)aniline	147	0.239
	Pentacyclo[5.2.1.0(1,5).0(5,9).0(6,8)]decane	132	0.192
C_{11}	1-Heptanol, 2,4-diethyl-	172	0.343
	Cyclohexane, 1-ethyl-2-propyl-	154	0.962
	2-Isopropyl-5-methyl-1-heptanol	172	1.419
	1-Undecene, 11-chloro-	188	0.414
	Z-1,6-Undecadiene	152	0.299
	1,4-Methanonaphthalene, 1,4-dihydro-	142	0.125
C_{12}	Cyclohexane, 1,2,4-triethenyl-	162	0.139
	3-Dodecen-1-yne, (E)-	164	0.215
	1-Decene, 2,4-dimethyl-	168	2.860
	Cyclooctene, 5,6-diethenyl-, cis-	162	0.165
		162	0.480

	Cyclopropane, 1-(2-methylene-3-butenyl)-1-(1-methylenepropyl)-	168	11.000
	1-Undecene, 7-methyl-	160	0.251
	Cyclohexane, 1-ethenyl-3-methylene-5-(1-propenylidene)-	168	0.217
	3-Heptene, 2,2,3,5,6-pentamethyl-	201	0.193
	Naphthalene, 1,6-dimethyl-4-nitro-	175	0.216
	1H-Indole, 2-ethyl-2,3-dihydro-3,3-dimethyl-	168	0.513
	Cyclooctane, 1-methyl-3-propyl-	**Total**	**31.534**
C_{13}		182	0.145
	2-Undecene, 4,5-dimethyl-, [R*,R*-(E)]-	198	0.478
	Cyclododecanemethanol	200	0.374
	11-Methyldodecanol	182	0.153
C_{14}	Cyclopentane, 1-pentyl-2-propyl-	198	1.137
	Dodecane, 4,6-dimethyl-	202	0.367
	1(2H)-Naphthalenone, 2-(1,1-dimethylethyl)-3,4-dihydro-	214	0.597
	2-Hexyl-1-octanol	188	0.172
	Oct-3-ene-1,5-diyne, 3-t-butyl-7,7-dimethyl-	198	0.251
	Dodecane, 4,6-dimethyl-	213	0.334
	1-Tetradecanamine	232	0.228
C_{15}	Tetradecane, 1-chloro-	368	0.239
	Heptafluorobutyric acid, undecyl ester	226	0.318
	11-Dodecen-1-ol, 2,4,6-trimethyl-, (R,R,R)-	212	0.784
C_{16}	Dodecane, 2,6,11-trimethyl-	226	0.266
	2,6,10-Trimethyltridecane	254	0.314
	2-Propenoic acid, tridecyl ester	222	1.090
	Cyclohexane, 1,1'-(1,2-dimethyl-1,2-ethanediyl)bis-	237	0.307
C_{17}	Hexadecanenitrile	238	1.751
	4-Tetradecene, 2,3,4-trimethyl-	251	0.411
	Heptadecanenitrile	262	0.279
	3-(Hydroxy-phenyl-methyl)-2,3-dimethyl-octan-4-one	240	0.248
	Heptadecane	232	0.433
	Benzene, (1-methyldecyl)-	232	0.139
	Benzene, (1-pentylhexyl)-	232	0.581
	Benzene, (1-butylheptyl)-	232	0.415
C_{18}	Benzene, (1-propyloctyl)-	252	8.095
	Cyclohexane, 1,2,3,5-tetraisopropyl-	252	0.138
	Cyclohexane, 1,2,3,4,5,6-hexaethyl-	246	0.295
	Benzene, (1-methylundecyl)-	270	5.485
	1-Dodecanol, 2-hexyl-	250	0.856
	Cyclohexane, 1,1'-(1,2-dimethyl-1,2-ethanediyl)bis-	276	0.467
	Butyric acid, 2-phenyl-, 2-ethylhexyl ester	246	0.376
	Benzene, (1-pentylheptyl)-	246	0.368
C_{19}	Benzene, (1-butyloctyl)-	260	0.442
	Benzene, (1-pentyloctyl)-	260	0.216
C_{20}	Benzene, (1-butylnonyl)-	282	1.219
	Eicosane	282	0.331
	Hexadecane, 2,6,11,15-tetramethyl-	298	5.929
	1-Dodecanol, 2-octyl-	288	0.314
	3-Dodecene, 1-(benzyloxy)-4-methyl	**Total**	**36.344**
C_{21-35}		296	1.718
	Heneicosane	340	0.119

	Acetic acid, 3,7,11,15-tetramethyl-hexadecyl ester	350	0.218
	Undec-10-ynoic acid, dodecyl ester	354	0.253
	Dodecane, 1,1'-oxybis-	368	0.616
	Hexadecyl nonyl ether	352	0.116
	2-Methyltetracosane	350	0.148
	1-Cyclopentyleicosane	366	0.612
	Hexacosane	380	0.293
	2-Methylhexacosane	415	0.140
	Heptacosane, 1-chloro-	528	2.674
	Hexacosyl pentafluoropropionate	408	0.184
	2-methyloctacosane	506	0.450
	Octacosyl trifluoroacetate	450	0.906
	Dotriacontane	634	2.808
	Triacontyl heptafluorobutyrate	492	1.034
	Pentatriacontane	612	0.539
	Dotriacontyl pentafluoropropionate	508	0.114
C_{36+}	Hexacosyl nonyl ether	**Total**	**12.939**
		662	0.129
	Dotriacontyl heptafluorobutyrate	590	0.202
	Tetratriacontyl trifluoroacetate	640	1.241
	Tetratriacontyl pentafluoropropionate	690	4.562
	Tetratriacontyl heptafluorobutyrate	618	9.410
	Hexatriacontyl trifluoroacetate	647	0.777
	Octatriacontyl trifluoroacetate	914	0.148
	Tetrapentacontane, 1,54-dibromo-	758	2.005
	Tetrapentacontane	842	0.709
	Hexacontane	**Total**	**19.182**

Condensable product at 600°C

C Number	Compound	MW	Area %
C_5	D-Proline	115	0.192
C_6	2-Pentanone, 4-hydroxy-4-methyl-	116	0.207
	2,4-Hexadienenitrile	93	0.106
	Hexanedinitrile	108	0.283
C_7	Bicyclo[4.1.0]heptane, 2-chloro-	130	0.101
	Silane, chloromethylphenyl-	156	0.117
	6-Heptene-1-nitrile	109	0.302
	Heptanonitrile	111	0.220
	Benzonitrile	103	0.093
	Cyclopentanecarbonitrile, 3-methylene-	107	0.289
	4-Cyanocyclohexene	107	0.476
C_8	Bicyclo[3.2.0]hepta-3,6-diene-1-carbonitrile	117	0.185
C_9	Quinoline, 5,6,7,8-tetrahydro-	133	0.265
	2,6-Dimethylbenzonitrile	131	0.228
	Aziridine, 1,2-diisopropyl-3-methyl-, trans-	141	0.312
	2-Propen-1-amine, N,N-bis(1-methylethyl)-	141	0.214
	1,2-Dioxaspiro[4.5]decan-3-one, 4-methylene-	168	0.151
	4-Piperidinone, 2,2,6,6-tetramethyl-	155	1.037
	2-Methylindoline	133	0.233
	Quinoline, 1,2,3,4-tetrahydro-	133	0.343
C_{10}	Naphthalene	128	0.151
	1,5-Cyclooctadiene, 1-ethyl-	136	0.161

	2-(1-Methylcyclopropyl)aniline	147	0.211
	Octane, 3,3-dimethyl-	142	0.139
	1H-Indole-2-methanamine, 5-methyl-	160	0.186
	(2,4,6-Trimethylcyclohexyl) methanol	156	1.256
C_{11}	Benzene, pentyl-	148	0.349
	Naphthalene, 2-methyl-	142	0.111
	1-Undecene, 11-chloro-	188	0.196
	3-Methyl-2-(3-methylpentyl)-3-buten-1-ol	170	0.345
	1-Heptanol, 2,4-diethyl-	172	4.338
	2-Isopropyl-5-methyl-1-heptanol	172	0.871
	Cyclohexane, 1-ethyl-2-propyl-	154	0.991
C_{12}	1-Isopropyl-1,4,5-trimethylcyclohexane	168	0.155
	Cyclopropane, 1-(2-methylene-3-butenyl)-1-(1-methylenepropyl)-	162	0.429
	Cyclooctene, 5,6-diethenyl-, cis-	162	0.138
	3-Dodecen-1-yne, (E)-	164	0.317
	1-Undecene, 7-methyl-	168	5.143
	1-Decene, 2,4-dimethyl-	168	2.209
	Cyclooctane, 1-methyl-3-propyl-	168	0.660
		**Total**	**23.710**
C_{13}	2-Undecene, 4,5-dimethyl-, [R*,R*-(E)]-	182	0.160
	Benzene, heptyl-	176	0.151
	Cyclododecanemethanol	198	0.667
	Cyclohexane, 2-butyl-1,1,3-trimethyl-	182	0.159
	11-Methyldodecanol	200	0.763
	Cyclopentane, 1-pentyl-2-propyl-	182	0.109
C_{14}	Bicylo[4.1.0]heptane, 7-bicyclo[4.1.0]hept-7-ylidene-	188	0.283
	Dodecane, 4,6-dimethyl-	198	0.179
	1(2H)-Naphthalenone, 2-(1,1-dimethylethyl)-3,4-dihydro-	202	0.337
	2-Hexyl-1-octanol	214	0.657
	Dodecane, 4,6-dimethyl-	198	0.675
	Benzene, octyl-	190	0.123
	5,5-Dimethyl-1-phenyl-3-hexyne-1,2-diol	218	0.158
	Tetradecane, 1-chloro-	232	0.193
	10-Dodecen-1-ol, 7,11-dimethyl-	212	2.735
	Cyclopentane, 1-butyl-2-pentyl-	196	0.418
C_{15}	Dodecane, 2,6,11-trimethyl-	212	0.713
	1,4-Methanobenzocyclodecene, 1,2,3,4,4a,5,8,9,12,12a-decahydro-	202	0.098
	2,6,10-Trimethyltridecane	226	0.365
C_{16}	N,N'-Dibenzylideneethylenediamine	236	0.171
	2-Propenoic acid, tridecyl ester	254	0.268
	Hexadecane, 1,16-dichloro-	294	0.297
	Hexadecanenitrile	237	0.320
	1-Decanol, 2-hexyl-	242	3.095
C_{17}	Heptadecanenitrile	251	0.228
	4-Tetradecene, 2,3,4-trimethyl-	238	2.060
	Heptadecane	240	0.213
	Benzene, (1-methyldecyl)-	232	0.346
	7-Heptadecene, 17-chloro-	272	0.171
	Benzonitrile, 4-(4-butylcyclohexyl)-	241	0.149

	Benzene, (1-pentylhexyl)-	232	0.130
	Benzene, (1-butylheptyl)-	232	0.479
	Benzene, (1-propyloctyl)-	232	0.194
C_{18}	Propyl tetradecyl carbonate	300	0.215
	1-Dodecanol, 2-hexyl-	270	3.975
	Benzene, (2,3-dimethyldecyl)-	246	0.227
	Benzene, (1-pentylheptyl)-	246	0.224
	Benzene, (1-butyloctyl)-	246	0.345
	Benzene, (1-methylundecyl)-	246	0.261
	Cyclohexane, 1,2,3,5-tetraisopropyl-	252	14.811
	Ethanediamide, N-(2-ethoxyphenyl)-N'-(2-ethylphenyl)-	312	0.603
C_{19}	1,18-Nonadecadien-7,10-dione	292	0.335
	Benzene, (1-pentyloctyl)-	260	0.393
	Benzene, (1-butylnonyl)-	260	0.174
C_{20}	2-t-Butyl-6-(2-hydroxy-2-naphthalen-1-yl-ethyl)-[1,3]dioxin-4-one	326	0.158
	1-Hexadecanol, 3,7,11,15-tetramethyl-	298	0.100
	Hexadecane, 2,6,11,15-tetramethyl-	282	0.210
	1-Dodecanol, 2-octyl-	298	7.149
	Eicosane	282	0.488
		**Total**	**46.732**
C_{21-35}	Henicos-1-ene	294	0.089
	Heneicosane	296	1.380
	Dodecane, 1,1'-oxybis-	354	0.231
	Tetracosane	338	0.161
	Pentadecafluorooctanoic acid, hexadecyl ester	638	0.323
	2-Methyltetracosane	352	0.097
	1-Cyclopentyleicosane	350	0.155
	Hexadecyl nonyl ether	368	0.385
	Hexacosane	366	0.446
	Benzene, (1-ethyloctadecyl)-	358	0.407
	Heptacosane, 1-chloro-	414	0.162
	2-Methylhexacosane	380	1.364
	Docosyl octyl ether	438	0.106
	Octacosyl trifluoroacetate	506	0.621
	Triacontyl trifluoroacetate	534	2.001
	Triacontyl heptafluorobutyrate	634	2.961
	Hexacosyl nonyl ether	508	0.904
	Pentatriacontane	492	0.714
		**Total**	**12.507**
C_{36+}	Tetratriacontyl trifluoroacetate	590	0.368
	Tetratriacontyl pentafluoropropionate	640	1.359
	Tetratriacontyl heptafluorobutyrate	690	4.129
	Hexatriacontyl trifluoroacetate	618	6.923
	Octatriacontyl trifluoroacetate	646	0.994
	Tetracontane	562	0.516
	Tetrapentacontane	758	0.618
	Tetrapentacontane, 1,54-dibromo-	914	0.984
	Hexacontane	842	1.158
		**Total**	**17.050**

Condensable product at 700°C			
C Number	Compound	MW	Area %
C_5	D-Proline	115	1.182
	Pentanenitrile	83	0.390
C_6	3-Penten-2-one, 4-methyl-	98	0.125
	2-Pentanone, 4-hydroxy-4-methyl-	116	0.419
	5-Cyano-1-pentene	95	0.298
	Hexanenitrile	97	1.030
	Aniline	93	0.183
	1H-Pyrazole, 4,5-dihydro-5-propyl-	112	0.523
C_7	Cyclopropane, (1-methyl-1,2-propadienyl)-	94	0.130
	2-Norbornanone	110	0.661
	Cyclopentanecarbonitrile, 3-methylene-	107	0.612
	4-Cyanocyclohexene	107	1.263
	Benzonitrile	103	0.352
	Isoxazole, 3-butyl-5-chloro-	159	0.159
	6-Heptene-1-nitrile	109	0.861
	Heptanonitrile	111	0.597
	Bicyclo[4.1.0]heptane, 2-chloro-	130	0.163
C_8	9-Oxabicyclo[6.1.0]nonane, 1-methyl-, cis-	126	0.161
	2,7-Octadien-1-ol	126	0.173
	Ethylbenzene	106	0.164
	Styrene	104	0.619
	Cyclopentane, (1-methylethyl)-	112	0.423
C_9	2-Methylindoline	133	0.443
	4-Piperidinone, 2,2,6,6-tetramethyl-	155	1.725
	2-Propen-1-amine, N,N-bis(1-methylethyl)-	141	0.324
	Nonane, 1-chloro-	162	0.992
	Cyclohexene, 3,5,5-trimethyl-	124	0.097
	2,4-Dimethyl-1-heptene	126	2.717
	2-Bromononane	206	0.254
	1,3,5-Cycloheptatriene, 7-ethyl-	120	0.326
	Indane	118	0.157
	Piperidin-4-one, 1-ethyl-2,3-dimethyl-	155	0.408
	Quinoline, 1,2,3,4-tetrahydro-	133	0.692
	2,6-Dimethylbenzonitrile	131	0.277
	Cyclopropanamine, 2-phenyl-, trans-	133	0.389
	1-Benzyl-1H-1,2,4-triazole	159	0.201
C_{10}	Naphthalene	128	0.188
	(2,4,6-Trimethylcyclohexyl) methanol	156	1.594
	Octane, 3,3-dimethyl-	142	0.530
	2,6-Octadien-1-ol, 2,7-dimethyl-	154	0.365
	trans, trans-Octa-2,4-dienyl acetate	168	0.104
	2H-Pyran, 3,6-dihydro-4-methyl-2-(2-methyl-1-propenyl)-	152	0.357
	2-(1-Methylcyclopropyl)aniline	147	0.366
	1,5-Cyclooctadiene, 1-ethyl-	136	0.248
C_{11}	Cyclohexane, 1-ethyl-2-propyl-	154	0.251
	1-Heptanol, 2,4-diethyl-	172	5.515
	2-Isopropyl-5-methyl-1-heptanol	172	0.663
	Pent-3-ene-2-one, 3-phenyl-, oxime	175	0.213

	Bicyclo[3.1.1]hept-2-ene-2-ethanol, 6,6-dimethyl-	166	0.221
	2-Decene, 4-methyl-, (Z)-	154	0.117
	Benzene, pentyl-	148	0.444
	1H-Indene, 2,3-dimethyl-	144	0.103
	3-Methyl-2-(3-methylpentyl)-3-buten-1-ol	170	0.333
C_{12}	1,5,7-Octatrien-3-ol, 2,6-dimethyl-	164	0.363
	Trichloroacetic acid, 6-ethyl-3-octyl ester	302	0.086
	1-Decene, 2,4-dimethyl-	168	4.073
	Cyclooctene, 5,6-diethenyl-, cis-	162	0.204
	1-Octanol, 2-butyl-	186	1.684
	Cyclopropane, 1-(2-methylene-3-butenyl)-1-(1-methylenepropyl)-	162	0.460
	Cyclooctane, 1-methyl-3-propyl-	168	1.629
	Dodecane, 1-chloro-	204	0.292
	1-Undecene, 7-methyl-	168	0.730
		**Total**	**40.623**
C_{13}	2-Undecene, 4,5-dimethyl-, [R*,R*-(E)]-	182	0.224
	11-Methyldodecanol	200	4.921
	Cyclododecanemethanol	198	0.789
	Cyclopropane, 1-(2-chloro-1-hexen-1-ylideno)-2,2,3,3-tetramethyl-	212	0.114
C_{14}	10-Dodecen-1-ol, 7,11-dimethyl-	212	0.228
	Tetradecane, 1-chloro-	232	0.204
	Bicylo[4.1.0]heptane, 7-bicyclo[4.1.0]hept-7-ylidene-	188	0.288
	Dodecane, 4,6-dimethyl-	198	0.901
	1(2H)-Naphthalenone, 2-(1,1-dimethylethyl)-3,4-dihydro-	202	0.460
	2-Hexyl-1-octanol	214	0.715
C_{15}	(3R,3aR,4aR,8aS,9aR)-3,8a-Dimethyl-5-methylenedecahydronaphtho[2,3-b]furan-2(3H)-one	234	0.104
	1,4-Methanobenzocyclodecene, 1,2,3,4,4a,5,8,9,12,12a-decahydro-	202	0.119
	(1aS,4aS,8aR)-4a,8,8-Trimethyl-2-methylene-1,1a,2,4a,5,6,7,8-octahydrocyclopropa[d]naphthalene	202	0.150
	Dodecane, 2,6,11-trimethyl-	212	0.250
	2,10-Dodecadien-1-ol, 3,7,11-trimethyl-, (Z)-	224	0.089
C_{16}	Cyclohexane, 1,1'-(1,2-dimethyl-1,2-ethanediyl)bis-	222	1.795
	2,6,10-Trimethyltridecane	226	0.169
	Hexadecanenitrile	237	0.357
	2-Propenoic acid, tridecyl ester	254	0.217
	Cyclohexadecane	224	0.110
	N,N'-Dibenzylideneethylenediamine	236	0.192
	Oxalic acid, 2-ethylhexyl isohexyl ester	286	0.517
	1-Decanol, 2-hexyl-	242	0.630
C_{17}	Ethanone, 1-phenyl-, o-(4-coumarinyl)oxime	279	0.109
	Heptadecanenitrile	251	0.214
	4-Tetradecene, 2,3,4-trimethyl-	238	0.128
	Benzene, (1-pentylhexyl)-	232	0.148
	Benzene, (1-butylheptyl)-	232	0.498
	Benzene, (1-propyloctyl)-	232	0.215
	Heptadecane	240	0.215
	Benzene, (1-methyldecyl)-	232	0.390

	Benzene, (1-ethylnonyl)-	232	0.426
C_{18}	Geranic acid, 2-Phenylethyl ester	272	0.231
	Benzene, (2,3-dimethyldecyl)-	246	0.293
	Benzene, (1-methylundecyl)-	246	0.274
	Cyclohexane, 1,2,3,5-tetraisopropyl-	252	9.744
	Triphenylphosphine oxide	278	0.418
	1-Decanol, 2-octyl-	270	0.250
	1-Dodecanol, 2-hexyl-	260	3.860
	Propyl tetradecyl carbonate	300	0.112
	Benzene, (1-pentylheptyl)-	246	0.263
	Benzene, (1-butyloctyl)-	246	0.336
C_{19}	Benzene, (1-propyldecyl)-	260	0.315
	Benzene, (1-pentyloctyl)-	260	0.404
	Benzene, (1-butylnonyl)-	260	0.199
	Benzene, (1-methyldodecyl)-	260	0.102
C_{20}	Eicosane	282	0.535
	2-t-Butyl-6-(2-hydroxy-2-naphthalen-1-yl-ethyl)-[1,3]dioxin-4-one	326	0.165
	Hexadecane, 2,6,11,15-tetramethyl-	282	0.227
	1-Dodecanol, 2-octyl-	298	7.118
		**Total**	**40.733**
C_{21-35}	Heneicosane	296	1.389
	Carbonic acid, octadecyl prop-1-en-2-yl ester	354	0.296
	Acetamide, 2,2-diphenyl-N-(3,3,5-trimethylcyclohexyl)-	335	0.178
	Hexadecyl nonyl ether	368	1.850
	Docosyl heptafluorobutyrate	522	0.239
	Hexacosane	366	0.402
	2-Methylhexacosane	380	0.706
	Carbonic acid, decyl hexadecyl ester	426	0.357
	Ditetradecyl ether	410	0.150
	Octacosyl trifluoroacetate	506	0.090
	Triacontyl pentafluoropropionate	584	1.032
	Triacontyl heptafluorobutyrate	634	0.934
	Hexacosyl nonyl ether	508	0.286
C_{36+}		**Total**	**7.909**
	Triacontyl heptafluorobutyrate	634	0.319
	Pentatriacontane	492	0.743
	Tetratriacontyl pentafluoropropionate	640	0.939
	Tetratriacontyl heptafluorobutyrate	690	1.268
	Hexatriacontyl trifluoroacetate	618	2.352
	Octatriacontyl trifluoroacetate	646	2.977
	Tetracontane	562	0.436
	Tetrapentacontane, 1,54-dibromo-	914	1.422
	Hexacontane	842	0.279
		**Total**	**10.735**

Condensable product at 800°C

C Number	Compound	MW	Area %
C_5	D-Proline	115	0.338
	Urea, N,N-diethyl-	116	0.763
C_6	2-Pentanone, 4-hydroxy-4-methyl-	116	0.310
	4-Pentenenitrile, 2-methylene-	93	0.108

	Aniline	93	0.154
C_7	6-Heptene-1-nitrile	109	0.390
	Heptanonitrile	111	0.300
	Benzonitrile	103	0.114
	Cyclopentanecarbonitrile, 3-methylene-	107	0.417
	4-Cyanocyclohexene	107	0.589
C_8	3-Cyclohexen-1-nitrile, 6-methyl-	121	0.237
	Cyclopropene, 1-methyl-3-(2-methylcyclopropyl)-	108	0.233
	Bicyclo[3.2.0]hepta-3,6-diene-1-carbonitrile	117	0.239
C_9	Cyclopentanecarbonitrile, 3-(1-methylethylidene)-	135	0.209
	Spiro[cyclopropane-1,2'-[6.7]diazabicyclo[3.2.2]non-6-ene]	150	0.150
	2-Methylindoline	133	0.317
	2-Propen-1-amine, N,N-bis(1-methylethyl)-	141	0.499
	4-Piperidinone, 2,2,6,6-tetramethyl-	155	2.352
	Piperidin-4-one, 1-ethyl-2,3-dimethyl-	155	0.441
	1,6-Heptadiene, 3,5-dimethyl-	124	0.402
	Quinoline, 1,2,3,4-tetrahydro-	133	0.864
	Cyclobutene, 1-cyclopropyl-2-ethoxy-3,3-difluoro-	174	0.388
	2,6-Dimethylbenzonitrile	131	0.304
	1-Benzyl-1H-1,2,4-triazole	159	0.260
	9-Oxabicyclo[6.1.0]nonane, 1-methyl-, cis-	140	0.177
C_{10}	(2,4,6-Trimethylcyclohexyl) methanol	156	1.462
	Decane, 1-iodo-	268	0.206
	Benzenesulfonamide, N-butyl-	213	0.468
	N-Phenylpyrrolidine	147	0.491
	Naphthalene	128	0.246
	2-(1-Methylcyclopropyl)aniline	147	0.342
	Octane, 3,3-dimethyl-	142	0.161
	1H-Indole, 2,3-dimethyl-	145	0.108
	1H-Indole-2-methanamine, 5-methyl-	160	0.221
C_{11}	2-Isopropyl-5-methyl-1-heptanol	172	5.051
	Cyclohexane, 1-ethyl-2-propyl-	154	1.522
	1-Heptanol, 2,4-diethyl-	172	0.132
	1-Undecene, 11-chloro-	188	0.248
	1H-Indene, 1,3-dimethyl-	144	0.112
	Naphthalene, 1,2-dihydro-3-methyl-	144	0.134
	Naphthalene, 2-methyl-	142	0.134
C_{12}	3-Dodecen-1-yne, (E)-	164	0.177
	1-Decene, 2,4-dimethyl-	168	2.266
	Cyclooctene, 5,6-diethenyl-	162	0.233
	Cyclopropane, 1-(2-methylene-3-butenyl)-1-(1-methylenepropyl)-	162	0.529
	1,5,9-Cyclododecatriene, (E,Z,Z)-	162	0.165
	Cyclooctane, 1-methyl-3-propyl-	168	0.296
	2-Bromo dodecane	248	0.133
		**Total**	**25.392**
C_{13}	Naphthalene, 1,2-dihydro-1,1,6-trimethyl-	172	0.223
	Decane, 5-ethyl-5-methyl-	184	0.225
	Benzene, (3,3-dimethyl-4-pentenyl)-	174	0.122
	2-Undecene, 4,5-dimethyl-, [R*,R*-(E)]-	182	0.196
	11-Methyldodecanol	200	7.588

	Benzene, heptyl-	176	0.248
	Cyclododecanemethanol	198	0.713
	Cyclopentane, 1-pentyl-2-propyl-	182	0.194
C_{14}	2-Hexyl-1-octanol	214	0.727
	4-Piperidinamine, 1,2,5-trimethyl-N-phenyl-	218	0.239
	Dodecane, 4,6-dimethyl-	198	0.963
	10-Dodecen-1-ol, 7,11-dimethyl-	212	2.981
	Tetradecane, 1-chloro-	232	0.249
C_{15}	(3R,3aR,4aR,8aS,9aR)-3,8a-Dimethyl-5-methylenedecahydronaphtho[2,3-b]furan-2(3H)-one (3.alpha.,3a.alpha.,4a.beta.,8a.alpha.,9a.alpha.)-	234	0.110
C_{16}	2,6,10-Trimethyltridecane	226	0.226
	Hexadecanenitrile	237	0.320
	1-Decanol, 2-hexyl-	242	6.623
	Cyclohexane, 1,1'-(1,2-dimethyl-1,2-ethanediyl)bis-	222	0.329
	2-Propenoic acid, tridecyl ester	254	0.215
	Cyclobuta[1,2:3,4]dicyclooctene, 1,2,5,6,6a,6b,7,8,11,12,12a,12b-dodecahydro-, (6a.alpha.,6b.alpha.,12a.alpha.,12b.beta.)-	216	0.147
	Oxalic acid, 2-ethylhexyl isohexyl ester	286	0.556
	1-Hexadecanesulfonyl chloride	324	0.452
C_{17}	4-Tetradecene, 2,3,4-trimethyl-	238	1.703
	Undecane, 3-cyclohexyl-	238	0.189
	n-Heptadecanol-1	256	0.407
	(S,Z)-Heptadeca-1,9-dien-4,6-diyn-3-ol	244	0.200
	7-Heptadecene, 1-chloro-	272	0.160
	Benzene, (1-pentylhexyl)-	232	0.161
	Benzene, (1-butylheptyl)-	232	0.582
	Benzene, (1-propyloctyl)-	232	0.254
	Benzene, (1-ethylnonyl)-	232	0.511
	Heptadecane	240	0.273
	Benzene, (1-methyldecyl)-	232	0.488
	Heptadecanenitrile	251	0.382
C_{18}	Benzene, (1-pentylheptyl)-	246	0.325
	Benzene, (1-butyloctyl)-	246	0.428
	1-Dodecanol, 2-hexyl-	270	9.367
	Benzene, (1,3,3-trimethylnonyl)-	246	0.296
	2-Pentyl-2,3,4,9-tetrahydro-1H-fluoren-9-ol	256	0.215
	Benzene, (1-methylundecyl)-	246	0.367
	Cyclohexane, 1,2,3,5-tetraisopropyl-	252	10.630
	1,3-Dimethyl-(3,7-dimethyloctyl)cyclohexane	252	0.387
C_{19}	Malonic acid, 2-chloropropyl tridecyl ester	362	0.234
	2-Methyloctadecan-7,8-diol	300	0.406
	7-Hexyltridecan-1-ol	284	0.207
	Benzene, (1-pentyloctyl)-	260	0.493
	Benzene, (1-butylnonyl)-	260	0.241
	Benzene, (1-propyldecyl)-	260	0.384
	Benzene, (1-ethylundecyl)-	260	0.384
C_{20}	Benzene, (1-methyldodecyl)-	260	0.160
	10-Methylnonadecane	282	0.337
	Hexadecane, 2,6,11,15-tetramethyl-	282	0.394

	1-Dodecanol, 2-octyl-	298	3.817
		**Total**	**58.030**
C_{21-35}	Nonadecyl trifluoroacetate	380	0.128
	Pentadecane, 8-hexyl-	296	0.145
	Heneicosane	296	0.943
	Nonadecyl pentafluoropropionate	430	0.191
	Docosane	310	0.596
	Nonyl tetradecyl ether	340	0.609
	11-Methyltricosane	338	0.219
	2-Methyltetracosane	352	0.114
	Hexacosane, 1-iodo-	492	0.252
	Hexacosane	366	0.527
	Triacontane, 1-iodo-	548	0.201
	Octacosyl trifluoroacetate	506	0.154
	Dotriacontane, 1-iodo-	576	0.647
	Triacontyl heptafluorobutyrate	634	1.670
		**Total**	**6.396**
C_{36+}	Hexacosyl nonyl ether	508	0.222
	Tetratriacontyl pentafluoropropionate	640	1.010
	Hexatriacontyl trifluoroacetate	618	3.900
	Octatriacontyl trifluoroacetate	646	2.090
	Octatriacontyl pentafluoropropionate	696	0.209
	Tetratetracontane	618	0.684
	Tetrapentacontane, 1,54-dibromo-	914	1.574
	Tetrapentacontane	758	0.378
	Hexacontane	842	0.116
		**Total**	**10.181**

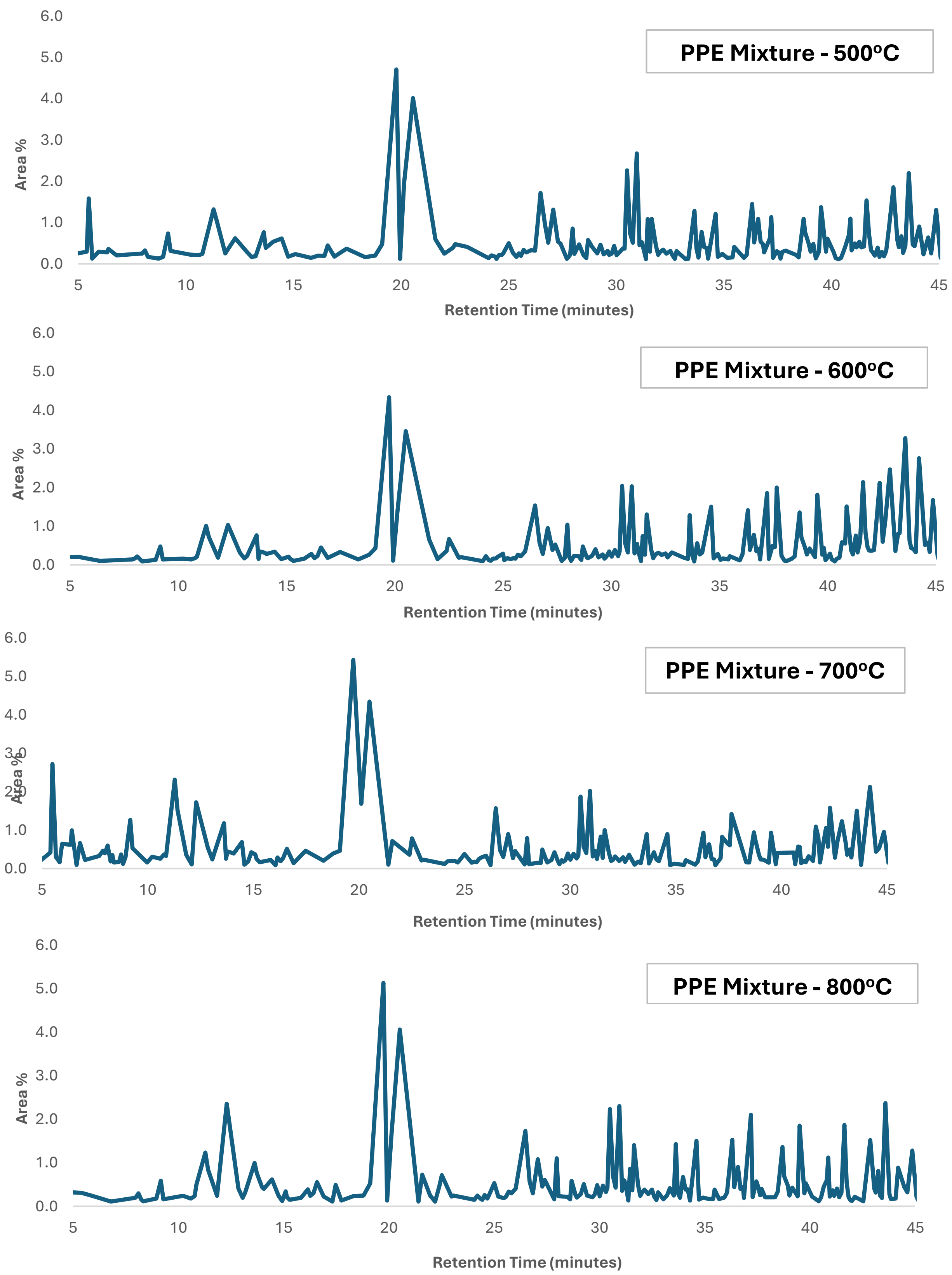

***Figure S1.* –** *GC-MS Chromatograms for PPE Mixture at 500, 600, 700, and 800°C*

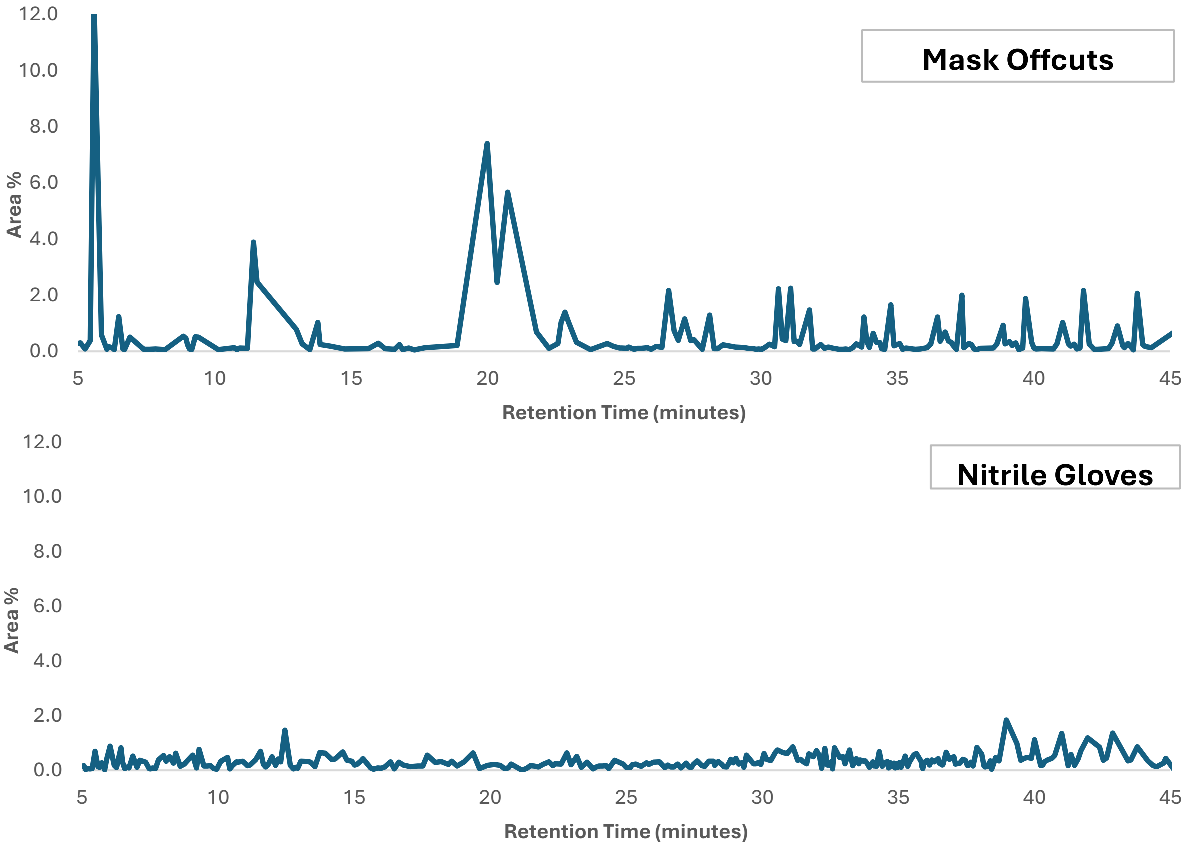

***Figure S2.* –** *GC-MS Chromatograms for Mask Offcuts and Nitrile Gloves at 700°C*

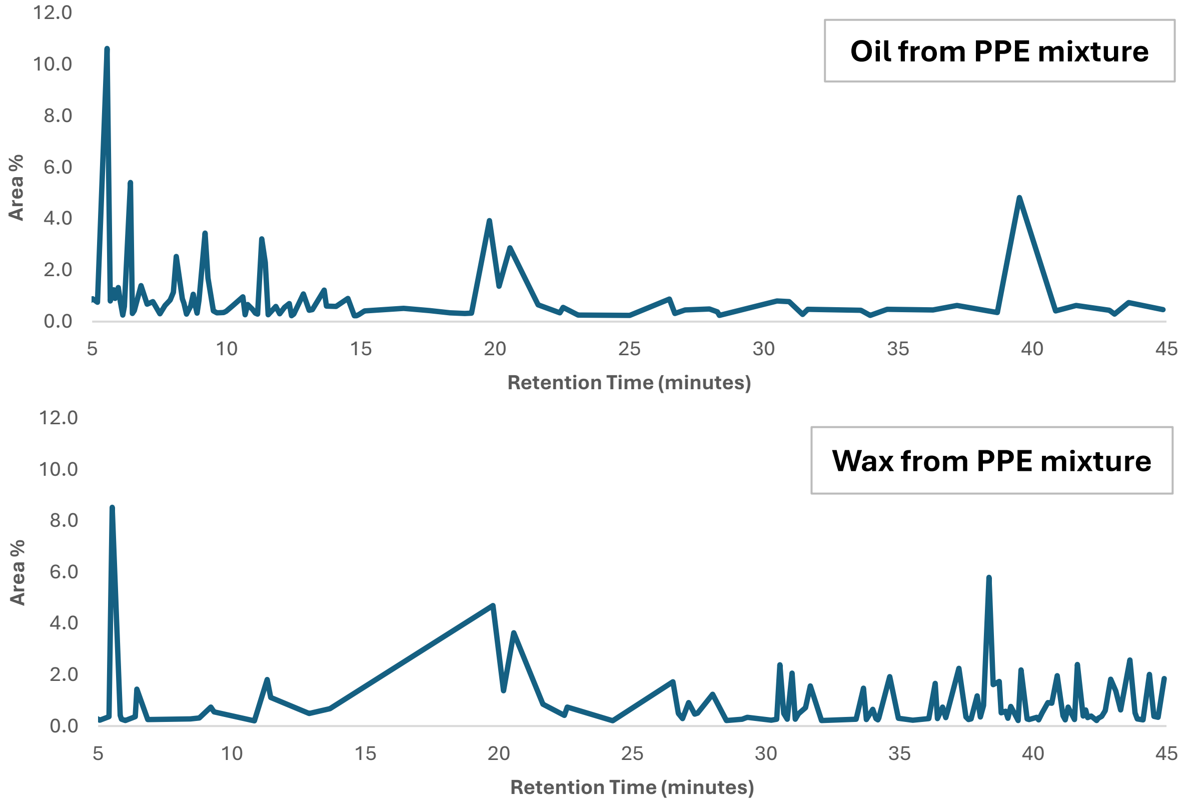

***Figure S3.* –** *GC-MS Chromatograms for PPE Mixture from Auger reactor at 500°C*

Table S2

Composition (GC-MS, Area %) of the condensable product obtained through thermal pyrolysis of the Mask offcuts and Nitrile gloves, and PPE mixture using the scale-up Auger reactor

Mask Offcuts at 700°C			
**C Number**	**Compound**	**MW**	**Area %**
C_7	Sulfone, methyl phenyl	156	1.398
C_8	4-Methyl-2-heptene	112	0.213
	Heptane, 4-methyl-	114	0.414
	1,5-Hexadiene, 2,5-dimethyl-	110	0.145
	o-Xylene	106	0.070
C_9	Mesitylene	120	0.068
	7-Methylbicyclo[4.2.0]octane	124	0.503
	Cyclohexane, 1,3,5-trimethyl-	126	0.968
	Cyclopropane, 1,1-dimethyl-2-(2-methyl-1-propenyl)-	124	0.171
	1,2,4,4-Tetramethylcyclopentene	124	0.117
	1-Octene, 2-methyl-	126	0.063
	1-Hexene, 3,3,5-trimethyl-	126	0.300
	Heptane, 2,4-dimethyl-	128	0.089
C_{10}	2,6-Dimethyl-6-nitro-2-hepten-4-one	185	0.081
	2-Pentanone, 3-[(acetyloxy)methyl]-3,4-dimethyl-, (.+-.)-	186	1.233
	2,6-Octadiene, 2,4-dimethyl-	138	0.064
	1-Octene, 3,4-dimethyl-	140	0.071
	2-Methyl-1-nonene	140	0.075
	Heptane, 3-ethyl-5-methyl-	142	0.088
	Heptane, 2,5,5-trimethyl-	142	0.517
	Heptane, 2,5,5-trimethyl-	142	0.507
	(2,4,6-Trimethylcyclohexyl) methanol	156	1.812
	1,6-Octadiene, 5,7-dimethyl-, (R)-	138	0.056
C_{11}	Cyclohexane, 1-ethyl-2-propyl-	154	1.478
	1-Heptanol, 2,4-diethyl-	172	0.099
	2-Isopropyl-5-methyl-1-heptanol	172	8.221
	Cyclohexane, 2,4-diethyl-1-methyl-	154	0.368
	1-Decene, 4-methyl-	154	0.064
	1-Undecene	154	12.467
	2-Decene, 4-methyl-, (Z)-	154	1.235
C_{12}	5-Undecene, 8-methyl-, (E)-	168	0.081
	1-Undecene, 7-methyl-	168	0.438
	2-Decene, 2,4-dimethyl-	168	0.066
	1-Isopropyl-1,4,5-trimethylcyclohexane	168	0.113
	1-Decene, 2,4-dimethyl-	168	7.131
	4-Undecene, 8-methyl-, (Z)-	168	0.062
	Dichloroacetic acid, 6-ethyl-3-octyl ester	268	0.278
	Cyclooctane, 1-methyl-3-propyl-	168	2.133
		**Total**	**43.257**
C_{13}	2-Undecene, 4,5-dimethyl-, [R*,R*-(E)]-	182	0.439
	11-Methyldodecanol	200	8.221
C_{14}	10-Dodecen-1-ol, 7,11-dimethyl-	212	0.579
	Dodecane, 4,6-dimethyl-	198	0.454
	2-Hexyl-1-octanol	214	1.081
	Dodecane, 4,6-dimethyl-	198	0.199

	Oxirane, dodecyl-	212	0.132
C_{15}	Dodecane, 2,6,11-trimethyl-	212	0.245
	11-Dodecen-1-ol, 2,4,6-trimethyl-, (R,R,R)-	226	0.063
C_{16}	1-Decanol, 2-hexyl-	242	0.494
	11-Dodecen-5-one, 3,3,7,7-tetramethyl-	238	0.086
	Cyclohexane, 1,1'-(1,2-dimethyl-1,2-ethanediyl)bis-	222	2.856
	1-Decanol, 2-hexyl-	242	0.224
	2,6,10-Trimethyltridecane	226	0.125
C_{17}	4-Tetradecene, 2,3,4-trimethyl-	238	0.358
	Heptadecane	240	0.083
C_{18}	1-Dodecanol, 2-hexyl-	270	12.158
	Cyclohexane, 1,2,3,5-tetraisopropyl-	252	10.516
C_{19}	2-Pentadecanone, 6,10,14-trimethyl-	268	0.074
C_{20}	Heptadecane, 8-methyl-	254	0.092
	Nonadecane	268	0.068
	Octadecane, 1-(ethenyloxy)-	296	0.118
	1,19-Eicosadiene	278	2.434
	Eicosane	282	0.558
	1-Dodecanol, 2-octyl-	298	0.617
	Hexadecane, 2,6,10,14-tetramethyl-	282	0.080
		**Total**	**42.353**
C_{21-35}	Heneicosane	296	0.077
	1-Cyclopentyleicosane	350	0.082
	Hexadecyl nonyl ether	368	0.363
	Octadecyl octyl ether	382	0.075
	2-Methylhexacosane	380	0.386
	Hexacosyl trifluoroacetate	478	0.629
	Octacosyl pentafluoropropionate	556	0.222
	1-Octacosanol, 2,4,6,8-tetramethyl-, (all-R)-	466	0.072
	Triacontyl heptafluorobutyrate	634	1.540
		**Total**	**3.448**
C_{36+}	Hexacosyl nonyl ether	508	0.093
	Tetratriacontyl pentafluoropropionate	640	1.217
	Tetratriacontyl heptafluorobutyrate	690	1.941
	Hexatriacontyl trifluoroacetate	618	4.955
	Octatriacontyl trifluoroacetate	646	2.087
	Tetratetracontane	618	0.392
	Tetrapentacontane, 1,54-dibromo-	914	0.257
		**Total**	**10.942**

**Nitrile Gloves at 700°C**			
**C Number**	**Compound**	**MW**	**Area %**
C_3	Propanenitrile	55	0.079
C_4	2-Butenenitrile	67	0.278
	Isobutyronitrile	69	0.036
	Butanenitrile	69	0.236
	Propane, 2-isocyanato-	85	0.171
C_5	2-Pentenenitrile	81	0.087
	Methallyl cyanide	81	0.161
	Cyclopentanone	84	0.158
	2-Pentanone	86	0.011

	Thiophene, 2-methyl-	98	0.131
	Pyrrolidine, 2-(hydroxymethyl)-	101	0.068
	1-Formyl-3-methylaziridine-2-carbonitrile	110	0.112
C_6	1-Pentyne, 4-methyl-	82	0.027
	4-Pentenenitrile, 2-methylene-	93	0.523
	Aniline	93	0.268
	5-Cyano-1-pentene	95	0.271
	3-Penten-2-one, 4-methyl-	98	0.195
	2,3-Diazabicyclo[2.2.2]oct-2-ene	110	0.064
	Butanedinitrile, 2,3-diamino-2,3-dimethyl-	138	0.139
C_7	Toluene	92	0.923
	1,3,5-Hexatriene, 3-methyl-, (Z)-	94	0.118
	Cyclopentene, 3-ethenyl-	94	0.127
	3-Methylenecyclohexene	94	0.125
	Benzonitrile	103	0.629
	Cyclopentanecarbonitrile, 3-methylene-	107	0.143
	4-Cyanocyclohexene	107	0.765
	Benzylamine	107	0.165
	2-Cyano-1-hexene	109	0.103
	6-Heptene-1-nitrile	109	0.877
	Cyclohexanecarbonitrile	109	0.552
	Heptanonitrile	111	0.498
	Cyclopentanone, 2-ethyl-	112	0.294
	Propanenitrile, 3-(diethylamino)-	126	0.043
	Hexane, 1-chloro-5-methyl-	134	0.138
	Sulfone, methyl phenyl	156	0.642
	1H-1,2,3,4-Tetrazole-1,5-diamine, N'1'-[(E)-(1-methyl-1H-pyrrol-2-yl)methylidene]-	191	0.068
	5-Methoxybenzo[1,2,5]selenadiazole	214	0.165
	Stannane, triethenylmethyl-	216	0.145
C_8	Bicyclo[4.2.0]octa-1,3,5-triene	104	0.828
	o-Xylene	106	0.302
	Ethylbenzene	106	0.392
	Octa-2,4,6-triene	108	0.126
	Cyclopentene, 1,2-dimethyl-4-methylene-	108	0.082
	Bicyclobutylidene	108	0.080
	Cyclohexene, 4-ethenyl-	108	0.844
	1,3,6-Heptatriene, 5-methyl-	108	0.131
	2,3-Dimethyl-cyclohexa-1,3-diene	108	0.113
	Cyclopentane, cyclopropylidene-	108	0.032
	1,5-Hexadiene, 2,5-dimethyl-	110	0.107
	4-Ethylcyclohexene	110	0.051
	1-Octene	112	0.088
	Octane	114	0.063
	Bicyclo[3.2.0]hepta-3,6-diene-1-carbonitrile	117	0.493
	Benzonitrile, 2-methyl-	117	0.697
	5H-1-Pyrindine, 6,7-dihydro-	119	0.196
	1-Cyclohexene-1-acetonitrile	121	0.091
	Octanenitrile	125	0.189
	3-Octyn-1-ol	126	0.217
	Cyclohexanemethanol, 2-methyl-	128	0.178

	Octanedinitrile	136	0.121
	1,2,3-Trimethylpiperidin-4-one	141	0.242
	Cyclooctene, 5-chloro-	144	0.331
	1,5-Cyclooctadiene, 3-bromo-	186	0.157
	2-Chloro-4,6-dimethoxyaniline	187	0.278
	9-Borabicyclo[3.3.1]nonane, 9-bromo-	200	0.550
	Stannane, tetraethenyl-	228	0.050
	Iron, tricarbonyl-chloro-.eta.-3-pentenyl-	244	0.134
	Molybdenum, tricarbonylchloro(.eta.5-2,4-cyclopentadien-1-yl)-	282	0.138
C_9	1-Propyne, 3-phenyl-	116	0.479
	Indane	118	0.334
	Benzene, (1-methylethyl)-	120	0.366
	1,3,5-Cycloheptatriene, 7-ethyl-	120	0.380
	Benzene, 1,2,4-trimethyl-	120	0.347
	Mesitylene	120	0.177
	Norbornane, 2-ethylidene-	122	0.048
	Cyclohexane, cyclopropylidene-	122	0.068
	2-Nonyne	124	0.171
	Nonane	128	0.071
	p-Ethylbenzonitrile	131	0.368
	1H-Indole, 5-methyl-	131	0.312
	2,6-Dimethylbenzonitrile	131	0.300
	2-Methylindoline	133	0.328
	Quinoline, 1,2,3,4-tetrahydro-	133	0.676
	Cyclopropanamine, 2-phenyl-, trans-	133	0.313
	3-Phenylpropanol	136	0.092
	8-Nonene-1-nitrile	137	0.418
	3-Cyclohexene-1-propanal	138	0.303
	4-Piperidinone, 2,2,6,6-tetramethyl-	155	1.469
	Ethinamate	167	0.059
	2-Chloro-4-piperidinopyrimidine	197	0.195
C_{10}	Naphthalene	128	0.336
	1H-Indene, 3-methyl-	130	0.645
	Benzene, (1-methyl-2-propynyl)-	130	0.626
	1H-Indene, 2,3-dihydro-4-methyl-	132	0.301
	Indan, 1-methyl-	132	0.219
	5,8-Dimethylenebicyclo[2.2.2]oct-2-ene	132	0.048
	Benzene, 1,4-diethyl-	134	0.055
	Benzene, n-butyl-	134	0.307
	Limonene	136	0.019
	Bicyclo[4.2.0.]octane, 6,7-dimethyl	138	0.085
	1H-Indole, 1,3-dimethyl-	145	0.244
	2-(1-Methylcyclopropyl)aniline	147	0.347
	Naphthalen-4a,8a-imine, 1,4,5,8-tetrahydro-	147	0.876
	N-Phenylpyrrolidine	147	0.550
	Benzene, [(2-propenyloxy)methyl]-	148	0.054
	Bicyclo[6.1.0]non-4-ene-9-carbaldehyde	150	0.165
	Bicyclo[3.1.1]heptan-3-one, 2,6,6-trimethyl-, (1.alpha.,2.beta.,5.alpha.)-	152	0.395
	(8R,S)-8-Hydroxymethyl-cis-bicyclo[4.3.0]-3-nonene	152	0.196

	1-Octanol, 3,7-dimethyl-	158	0.121
	3-Isoquinolinamine, 4-methyl-	158	0.169
	1-Hexyne, 6-(1-ethoxyethoxy)-	170	0.021
	1,5-Naphthalenediol, decahydro-	170	0.114
	10-Chlorotricyclo[4.2.1.1(2,5)]deca-3,7-dien-9-ol	182	0.226
	Anthranilic acid, TMS derivative	209	0.498
	3-p-Tolyl-isoxazole-4,5-dione dioxime	219	0.271
C_{11}	Naphthalene, 1-methyl-	142	0.217
	Naphthalene, 1,2-dihydro-3-methyl-	144	0.425
	1H-Indene, 1,3-dimethyl-	144	0.484
	Benzene, 1-methyl-3-(1-methyl-2-propenyl)-	146	0.153
	1H-Indene, 2,3-dihydro-1,6-dimethyl-	146	0.189
	7-Methyl-1,2,3,5,8,8a-hexahydronaphthalene	148	0.041
	Benzene, (1-methylbutyl)-	148	0.184
	1,7-Octadiene, 2,3,3-trimethyl-	152	0.316
	Z-1,6-Undecadiene	152	0.879
	1-Naphthalenecarbonitrile	153	0.195
	Acetonitrile, (3,5,5-trimethyl-2-cyclohexen-1-ylidene)-, (Z)-	161	0.125
	Tricyclo[7.1.0.0[1,3]]decane-2-carbaldehyde	164	0.154
	10-Undecenenitrile	165	0.093
	Bicyclo[3.1.1]hept-2-ene-2-ethanol, 6,6-dimethyl-	166	0.423
	Pentamethylbenzenesulphonamide	227	0.160
	Nonane, 2-bromo-5-ethyl-	234	0.844
	3-Bromo-7-methoxy-1-[2-tetrahydropyranyl]pyrazolo[4,3-d]pyrimidine	312	0.376
C_{12}	Naphthalene, 1,7-dimethyl-	156	0.158
	Benzene, 2,5-cyclohexadien-1-yl-	156	0.162
	Naphthalene, 1,3-dimethyl-	156	0.368
	Naphthalene, 2,6-dimethyl-	156	0.301
	Benzene, (1,1-dimethyl-2-butynyl)-	158	0.185
	1,2,3-Trimethylindene	158	0.223
	Benzene, 1-hexynyl-	158	0.028
	(1-Methylpenta-2,4-dienyl)benzene	158	0.030
	Naphthalene, 1,2,3,4-tetrahydro-6,7-dimethyl-	160	0.066
	Cyclooctene, 5,6-diethenyl-, cis-	162	0.183
	3-Dodecen-1-yne, (E)-	164	0.229
	3-Dodecyne	166	0.062
	1-Naphthalenamine, N-ethyl-	171	0.309
	Quinoline, 4-propyl-	171	0.114
	13-Oxatetracyclo[4.4.1.1(7,10).1(9,11)]trideca-2,4-diene	174	0.061
	1,2-Epoxy-5,9-cyclododecadiene	178	0.119
	Tricyclo[7.3.0.0(2,6)]-8-dodecen-3-ol, (3R,S)-trans-anti-	178	0.246
	Tricyclo[5.2.2.1(2,6)]dodecan-12-ol	180	0.068
	5-Ethyl-5-methyl-2-phenyl-2-oxazoline	189	0.335
	3-Hydroxymethyl-4-(1-hydroxy-2-methylprop-2-enyl)toluene	192	0.108
	Naphthalene, 1,6-dimethyl-4-nitro-	201	0.275
	1-Cyclohexyl-3-hydroxydihydro-1H-furo[3,4-b]pyrrole-2,6(3H,4H)-dione	239	0.216
		**Total**	**40.096**
C_{13}	1-Isopropenylnaphthalene	168	0.316

	1,1'-Biphenyl, 2-methyl-	168	0.321
	Naphthalene, 2,3,6-trimethyl-	170	0.266
	Benzene, 1-methyl-4-[(1-methylethylidene)cyclopropyl]-	172	0.232
	(4-Methyl-1-methylenepent-4-enyl)benzene	172	0.151
	Benzene, heptyl-	176	0.315
	Cyclohexane, 1,2-diethenyl-4-(1-methylethylidene)-, cis-	176	0.195
	[1,1'-Biphenyl]-4-methanol	184	0.187
	Benzenemethanol, .alpha.-1-cyclohexen-1-yl-	188	0.146
	Tricyclo[6.3.0.0(1,5)]undec-2-en-4-one, 5,9-dimethyl-	190	0.186
	1-Ethyl-12-oxatetracyclo[5.2.1.1(2,6).1(9,11)]dodecane	192	0.147
	2,5-Dimethyl-1-[(2-hydroxymethyl)phenyl]pyrrole	201	0.283
	trans-7a-Ethoxycarbonyl-8-methoxy-bicyclo(4,3,0)non-2-ene		
	4-Aminobutyramide, N-methyl-N-[4-(1-pyrrolidinyl)-2-butynyl]-	224	0.341
	(+-)-5-(1-Acetoxy-1-methylethyl)-2-methyl-2-cyclohexen-1-	237	0.129
	one semicarbazone	267	0.976
C_{14}	9H-Fluorene, 1-methyl-	180	0.408
	1H-Indene, 3-ethyl-1-(1-methylethyl)-	186	0.091
	Biphenylene, 1,2,3,6,7,8,8a,8b-octahydro-4,5-dimethyl-	188	0.075
	1,3-Cyclohexadiene, 2,6,6-trimethyl-1-(3-methyl-1,3-butadienyl)-		
	Bicylo[4.1.0]heptane, 7-bicyclo[4.1.0]hept-7-ylidene-	188	0.210
	Benzene, octyl-	188	0.192
	2-Aminobiphenyl, N,N-dimethyl-	190	0.207
	(2-Methyl-3-biphenylyl)methanol	197	0.497
	2,5-Dimethyl-1-[(2-hydroxymethyl)phenyl]pyrrole	198	0.615
	2(1H)-Naphthalenone, 7-ethynyl-4a,5,6,7,8,8a-hexahydro-	201	0.242
	1,4a-dimethyl-, (1.alpha.,4a.beta.,7.beta.,8a.alpha.)-	202	0.087
	1,4-Methanobenzocyclodecene, 1,2,3,4,4a,5,8,9,12,12a-	202	0.172
	decahydro-	204	0.126
	2-Naphthalenemethanol, 8-ethenyl-3,4,4a,5,6,7,8,8a-octahydro-5-methylene-	204	0.753
	(3aR,6R,8aR)-7,7-Dimethyl-8-methylenehexahydro-1H-3a,6-methanoazulen-3(2H)-one	206	0.344
	Tricyclo[5.3.1.1(2,6)]dodecan-11-ol, 11-methyl-12-methylene-		
	(1R,4aS,6R,8aS)-8a,9,9-Trimethyl-1,2,4a,5,6,7,8,8a-octahydro-1,6-methanonaphthalen-1-ol	206	0.364
	2-[1-Naphthyl]-3-oxobutyronitrile	209	0.233
	Ethanone, 1,2-diphenyl-, oxime	211	0.112
	2-Methyl-7-phenyl-2,6-heptanediol	222	0.115
	2-[6-Methyl-2-pyridyl]methylenequinuclidine-3-one	228	0.042
	Benzonitrile, 4-(5-propyl-1,3-dioxan-2-yl)-, trans-	231	0.218
	Methyl 3-[3-(3-methoxy-3-oxopropyl)phenyl]propanoate	250	0.076
	Diglycolic acid, 2-ethylbutyl isobutyl ester	274	0.182
	Thiourea, N-(2,3-dimethylphenyl)-N'-[2-(1H-imidazol-5-yl)ethyl]-	274	0.085
C_{15}	1,7-Dimethyl-3-phenyltricyclo[4.1.0.0(2,7)]hept-3-ene	196	0.353
	Benzene, 1-methyl-3-[(4-methylphenyl)methyl]-	196	0.407
	Bicyclo[3.2.2]non-2-ene, 2-phenyl-	198	0.326

	4,4-Dimethyl-3-(3-methylbut-3-enylidene)-2-methylenebicyclo[4.1.0]heptane	202	0.120
	1,4-Methanobenzocyclodecene, 1,2,3,4,4a,5,8,9,12,12a-decahydro-	202	0.126
	Hexane, 2-phenyl-3-propyl-	204	0.304
	Benzene, nonyl-	204	0.340
	1,1,4,7-Tetramethyl-1a,2,3,4,6,7,7a,7b-octahydro-1H-cyclopropa[e]azulene	204	0.190
	Bicyclogermacrene	204	0.291
	Tricyclo[2.2.1.0(2,6)]heptane, 1,7-dimethyl-7-(4-methyl-3-pentenyl)-, (-)-	204	0.301
	5H-Dibenzo[a,d]cyclohepten-5-ol, 10,11-dihydro-	210	0.377
	Dodecane, 2,6,10-trimethyl-	212	0.016
	(1R,2R,4S,6S,7S,8S)-8-Isopropyl-1-methyl-3-methylenetricyclo[4.4.0.02,7]decan-4-ol	220	0.212
	(1R,1aR,2aS,5R,6R,6aS,7aS)-1,6,6a-trimethyldecahydro-1,2a-methanocyclopropa[b]naphthalen-5-ol	220	0.316
	3-(3-Aminophenyl)-1-phenyl-2-propen-1-one	223	0.409
	Glaucic acid	234	0.185
	Curcumenol	234	0.428
	5H-Cyclopropa[a]naphthalen-5-one, 1,1a,2,3,6,7,7a,7b-octahydro-3-hydroxy-1,1,7,7a-tetramethyl-	234	0.405
	Bicyclo[4.4.0]dec-5-ene, 1,5-dimethyl-3-hydroxy-8-(1-methylene-2-hydroxyethyl-1)-	236	0.126
	Butanoic acid, 3-[(1-phenylethyl-2-propynyl)oxy]	246	0.640
	Benzoic acid, 2,4-dinitro-, bicyclo[3.2.1]oct-6-yl ester	320	0.236
C_{16}	Cyclobuta[1,2:3,4]dicyclooctene, 1,2,5,6,6a,6b,7,8,11,12,12a,12b-dodecahydro-, (6a.alpha.,6b.alpha.,12a.alpha.,12b.beta.)-	216	0.235
	Benzene, (1-ethyloctyl)-	218	0.211
	1,11-Hexadecadiyne	218	0.288
	Benzene, (1-methylnonyl)-	218	0.331
	Benzene, (1-butylhexyl)-	218	0.233
	Benzene, decyl-	218	0.370
	11-Hexadecyn-1-ol	238	0.185
	7-Hexadecenal, (Z)-	238	0.301
	2,5-cyclohexadiene-1,4-dione, 2,5-bis(3-methyl-2-buten-1-yl)-	244	0.208
	Bicyclo[6.1.0]nonane-9,9-dicarbonitrile, 1-(1-cyclopenten-3-on-1-yl)-	254	0.315
	2-Propenoic acid, tridecyl ester	254	0.240
	Hydrazinecarboxamide, 2-(1,3-diphenyl-2-propenylidene)-	265	0.287
	Diethylmalonic acid, ethyl hept-4-yl ester	286	0.152
	3-Methylpyrazolo[(9-borabicyclo[3,3,1]nonyl)(diethylboryl]oxide	288	0.172
	Hexadecane, 1-iodo-	352	0.689
	Benzene, (1-propylheptyl)-	218	0.132
C_{17}	Tricyclo[5.2.1.0(2,6)]decane, 3-methylene-4-phenyl-	224	0.753
	Benzene, (1-pentylhexyl)-	232	0.275
	Benzene, (1-butylheptyl)-	232	0.399
	Benzene, (1-propyloctyl)-	232	0.445

	Benzene, (1-ethylnonyl)-	232	0.561
	Benzene, (1-methyldecyl)-	232	0.617
	Benzene, undecyl-	232	0.369
	Heptadecane	240	0.219
	Heptadecanenitrile	251	0.609
	7-(1-Chloro-2-phenyl-vinyl)-1-ethoxy-bicyclo[4.1.0]heptane	276	0.109
	Ethanone, 1-phenyl-, o-(4-coumarinyl)oxime	279	0.415
	Bicyclo[3.3.0]octa-2,6-diene-2-carbonitrile, 7-[(4-chlorophenyl)sulfonyl]-1,5-dimethyl-	333	0.505
	(cis-2,3,4,trans-6-Tetramethyl-3-cyclohexenyl)formaldehyde 2,4-dinitrophenylhydrazone	346	0.341
	Benzhydrazide, 4-methoxy-N2-(2-trifluoroacetylcyclohepten-1-yl)-	356	0.592
C_{18}	Hex-1-ene,2,5-diphenyl-	236	0.350
	Spiro[cyclobutane-1,1'(2'H)-phenanthrene], 3',4',4'a,9',10',10'a-hexahydro-4'a-methyl-, trans-	240	0.273
	Benzene, dodecyl-	246	0.479
	Benzene, (1-methylundecyl)-	246	0.608
	Benzene, (1-ethyldecyl)-	246	0.859
	Benzene, (1-pentylheptyl)-	246	0.648
	Benzene, (1-butyloctyl)-	246	0.737
	Benzene, (1-propylnonyl)-	246	0.648
	Cyclohexane, 1,2,3,5-tetraisopropyl-	252	0.443
	Octadecane	254	0.311
	Heptadecane, 2-methyl-	254	0.903
	2H-3,5a-Epoxynaphth[2,1-b]oxepin, dodecahydro-3,8,8,11a-tetramethyl-, [3R-	278	0.435
	(3.alpha.,5a.alpha.,7a.beta.,11a.alpha.,11b.beta.)]-	281	0.159
	Benzamide, N-[(3,4-dihydro-1H-2-benzopyran-1-yl)methyl]-4-methyl-		
	Oxazolo[4,3-a]isoquinolin-3-one, 1,5,6,10b-tetrahydro-1,10b-dimethyl-1-(3-pyridinyl)-	294	0.049
	1-[2-(5-Amino-tetrazol-1-yl)-acetylamino]-cyclohexanecarboxylic acid (2,6-dimethyl-phenyl)-amide	371	0.394
	Octadecane, 1-iodo-	380	0.169
C_{19}	Benzene, (1-pentyloctyl)-	260	0.715
	Benzene, (1-butylnonyl)-	260	0.529
	Benzene, (1-propyldecyl)-	260	0.803
	Benzene, (1-ethylundecyl)-	260	0.827
	Benzene, (1-methyldodecyl)-	260	0.745
	Tridecane, 6-cyclohexyl-	266	0.732
	Tridecane, 6-cyclohexyl-	266	0.497
	2-Pyrrolidinone, 4-(1H-1,3-benzimidazol-2-yl)-1-(2-ethylphenyl)-	305	0.118
	2-Pyrrolecarboxylic acid, 3,5-dimethyl-4-[1-(2-ethylphenylhydrazono)ethyl-, ethyl ester	327	0.261
C_{20}	Norethynodrel	298	0.262
	cis-5,8,11,14,17-Eicosapentaenoic acid	302	0.073
	1-Ethenyl(diphenyl)silyloxyhexane	310	0.068
	.3,14-Dihydroxy-18-nor-18-propylestra-1,3.5(10)-trien-17-one	314	0.324

	1-Phenanthrenecarboxylic acid, 7-ethenyl-		
	1,2,3,4,4a,4b,5,6,7,9,10,10a-dodecahydro-6-hydroxy-		
	1,4a,7-trimethyl-	318	0.308
	4-Chlordehydromethyltestosterone		
		334	0.152
	Heneicosane	**Total**	**40.181**
C_{21-35}	Pyrethrin II	296	3.442
	Pentadecane, 8-heptyl-	372	0.188
	Retinol, acetate	310	0.372
	Cyclodecacyclotetradecene, 14,15-didehydro-	328	0.249
	1,4,5,8,9,10,11,12,13,16,17,18,19,20-tetradecahydro-	296	0.435
	Pregnane-3,11,20,21-tetrol, cyclic 20,21-(methylboronate),		
	(3.alpha.,5.alpha.,11.beta.,20S)-	376	0.366
	1,4-benzenediamine, N4-(diphenylmethylene)-N1,N1-		
	diethyl-	328	0.581
	phenol, 4-[4,5-bis[4-(dimethylamino)phenyl]-4H-imidazol-		
	2-yl]-	398	0.637
	Hexanoic acid, octadecyl ester		
	Resibufogenin	368	0.108
	Tetracosane, 1-iodo-	384	0.163
	Hydrocortisone, bis(O-ethyloxime)	464	0.550
	Hexacosane, 1-iodo-	448	0.295
	Benzeneethanol, .alpha.,.alpha.,.beta.-triphenyl-	492	0.455
	(9Z,12Z)-Phenethyl octadeca-9,12-dienoate	350	0.291
	2-Methylhexacosane	384	0.173
	3-Methylhexacosane	380	0.755
	Cholest-5-en-3-ol (3.beta.)-, carbonochloridate	380	0.717
	(9Z,12Z,15Z)-3,7-Dimethyloct-6-en-1-yl octadeca-9,12,15-	448	0.279
	trienoate	416	0.077
	Bis(2-hydroxy-5-methyl-3-[1-		
	methylcyclohexyl]phenyl)methane	420	0.154
	2-methyloctacosane		
	Heptacosyl pentafluoropropionate	408	0.940
	Dotriacontane, 1-iodo-	542	0.343
		576	0.376
	Hexatriacontane	**Total**	**11.945**
C_{36+}	Octatriacontane, 1,38-dibromo-	506	2.535
	Lycopene	690	0.471
	.beta.,.Psi.-Carotene, 3',4'-didehydro-1',2'-dihydro-1',2'-	536	0.198
	dihydroxy-, (2'R)-	568	0.245
	Tetracontane		
	Fucoxanthin	562	2.223
	Tetratetracontane	658	0.266
		618	1.841
		**Total**	**7.778**

Mixture Oil at 500°C (Auger Reactor)			
C Number	Compound	MW	Area %
C_4	3-Butenenitrile	67	0.244
	Butanenitrile	69	0.286
C_6	5-Cyano-1-pentene	95	0.797
	Methanamine, N-[3-methyl-2-butenylidene]	97	4.022
	Hexanenitrile	97	1.323
	4-Penten-2-one, 4-methyl-	98	0.500
	3-Penten-2-one, 4-methyl-	98	0.689
	3-Chlorohexane	120	0.310
C_7	Benzonitrile	103	0.890
	Cyclopentanecarbonitrile, 3-methylene-	107	0.293
	Cyclohexen-1-carbonitrile	107	0.614
	o-Toluidine	107	0.475
	6-Heptene-1-nitrile	109	1.972
	Cyclohexanecarbonitrile	109	0.829
	Heptanonitrile	111	2.538
	Cyclopentanone, 2-ethyl-	112	0.775
	Piperidine, 2,3-dimethyl-	113	3.435
C_8	Ethylbenzene	106	0.896
	1,5-Hexadiene, 2,5-dimethyl-	110	0.389
	Hexane, 3-methyl-4-methylene-	112	0.253
	4-Methyl-2-heptene	112	0.838
	Heptane, 4-methyl-	114	2.316
	Bicyclo[3.2.0]hepta-3,6-diene-1-carbonitrile	117	0.589
	5H-1-Pyrindine, 6,7-dihydro-	119	0.705
	2(3H)-Furanone, 3-(dihydro-2(3H)-furanylidene)dihydro-	154	0.236
C_9	Indane	118	0.405
	Benzene, 2-propenyl-	118	0.342
	Benzene, 1-ethenyl-3-methyl-	118	0.712
	Benzene, (1-methylethyl)-	120	0.681
	1-Nonyne	124	0.664
	Cyclopropane, 1,1-dimethyl-2-(2-methyl-1-propenyl)-<br>7-Methylbicyclo[4.2.0]octane	124	0.803
	Cyclohexene, 3,5,5-trimethyl-	124	1.398
	2,3-Dimethyl-3-heptene, (Z)-	124	0.595
	Cyclohexane, 1,3,5-trimethyl-	126	0.885
	Octane, 2-methyl-	126	1.243
	Heptane, 2,4-dimethyl-	128	0.962
	Benzenamine, 2-(1-methylethenyl)-	128	0.755
	Benzenamine, 2-cyclopropyl-	133	0.472
	Quinoline, 1,2,3,4-tetrahydro-	133	0.896
	Bicyclo[3.1.1]heptan-2-one, 6,6-dimethyl-, (1R)-	133	0.320
	4-Piperidinone, 2,2,6,6-tetramethyl-	138	0.432
C_{10}	Naphthalene	155	0.230
	3a,6-Methano-3aH-indene, 2,3,6,7-tetrahydro-	128	0.228
	Bicyclo[4.1.0]heptane, 7-(1-methylethylidene)-	132	0.262
	3-Decen-1-yne, (Z)-	136	0.603
	Heptane, 2,5,5-trimethyl-	136	0.732
	2,3-Cycloheptenopyridine	142	1.696
	Bicyclo[6.1.0]non-4-ene-9-carbaldehyde	147	0.412

	(2,4,6-Trimethylcyclohexyl) methanol	150	0.845
C_{11}	2-Decene, 4-methyl-, (Z)-	156	1.825
	1-Undecene	154	1.646
	Cyclopropanecarboxaldehyde, 2-methyl-2-(4-methyl-3-pentenyl)-, trans-(.+-.)-	154	10.615
	2-Isopropyl-5-methyl-1-heptanol	166	0.324
C_{12}	Pentalene, octahydro-1,4-divinyl-	172	3.249
	Cyclopropane, 1-(2-methylene-3-butenyl)-1-(1-	162	0.346
	methylenepropyl)-	162	0.332
	1-Undecene, 8-methyl-	168	5.405
	1-Undecene, 7-methyl-	168	0.331
	1-Dodecene	168	0.274
	Cyclooctane, 1-methyl-3-propyl-	168	0.481
	1-Decene, 2,4-dimethyl-	168	5.519
	1-Undecene, 8-methyl-	168	0.357
	2-Decene, 2,4-dimethyl-	168	0.272
		**Total**	**73.757**
C_{13}	11-Methyldodecanol	200	5.299
	Dodecane, 4,6-dimethyl-	198	0.751
C_{14}	1(2H)-Naphthalenone, 2-(1,1-dimethylethyl)-3,4-dihydro-	202	0.429
	2-Hexyl-1-octanol	214	0.995
	3-(Benzyloxymethyl)hex-5-ene-1,2-diol	236	0.622
C_{15}	1,1'-Bicyclohexyl, 2-propyl-, cis-	208	0.289
	Cyclohexane, 1,1'-(1-methylethylidene)bis-	208	0.586
C_{16}	Cyclohexane, 1,1'-(1,2-dimethyl-1,2-ethanediyl)bis-	222	0.978
	Ethane, 1-(9-borabicyclo[3.3.1]non-9-yl)oxy-2-phenyl-	242	0.299
C_{17}	n-Heptadecanol-1	256	1.528
C_{18}	1-Dodecanol, 2-hexyl-	270	3.498
	2-Benzoyl-4,4-dimethyl-5-phenyl-4H-1,3-diazol-1-ium-1-olate	292	0.230
	Cyclohexane, 1,2,3,5-tetraisopropyl-	252	1.999
C_{20}	1,19-Eicosadiene	278	0.808
		**Total**	**18.310**
C_{21-35}	Squalene	410	4.816
	Bis(tridecyl) phthalate	530	0.288
	Triacontyl heptafluorobutyrate	634	0.466
		T**otal**	**5.571**
C_{36+}	Tetratriacontyl heptafluorobutyrate	690	1.149
	Hexatriacontyl trifluoroacetate	618	1.213
		**Total**	**2.362**

Mixture Wax at 500°C (Auger Reactor)			
**C Number**	**Compound**	**MW**	**Area %**
C_8	Styrene	104	0.366
	Ethylbenzene	106	0.271
	Benzene, 1,3-dimethyl-	106	0.220
C_9	7-Methylbicyclo[4.2.0]octane	124	0.248
	2,3-Dimethyl-3-heptene, (Z)-	126	0.224
	Cyclohexane, 1,3,5-trimethyl-	126	0.774
	2,4-Dimethyl-1-heptene	126	8.527
	(3H)Indazole, 3,3-dimethyl-	146	0.282
C_{10}	Octane, 3,3-dimethyl-	142	0.734
	Heptane, 2,5,5-trimethyl-	142	0.554
	(2,4,6-Trimethylcyclohexyl) methanol	156	0.888
C_{11}	2-Decene, 4-methyl-, (Z)-	154	0.316
	Cyclohexane, 1-ethyl-2-propyl-	154	2.507
	2-Isopropyl-5-methyl-1-heptanol	172	0.287
C_{12}	Benzene, (2,2-dimethylbutyl)-	162	0.646
	1-Undecene, 8-methyl-	168	1.439
	1-Decene, 2,4-dimethyl-	168	2.926
	1-Undecene, 7-methyl-	168	0.691
	Cyclooctane, 1-methyl-3-propyl-	168	1.662
	Cyclohexane, 1,5-diethyl-2,3-dimethyl-	168	0.642
	2-Dodecenal, (E)-	182	0.207
		**Total**	**24.411**
C_{13}	11-Methyldodecanol	200	4.697
C_{14}	10-Dodecen-1-ol, 7,11-dimethyl-	212	1.470
	2-Hexyl-1-octanol	214	1.266
C_{15}	Benzene, 1,1'-(1,3-propanediyl)bis-	196	0.262
	1,7-Dimethyl-4-(1-methylethyl)cyclodecane	210	0.358
C_{16}	Cyclohexane, 1,1'-(1,2-dimethyl-1,2-ethanediyl)bis-	222	1.560
	1-Decanol, 2-hexyl-	242	0.325
	Hexadecane, 1-chloro-	260	0.336
C_{17}	4-Tetradecene, 2,3,4-trimethyl-	238	1.927
C_{18}	Cyclohexane, 1,2,3,5-tetraisopropyl-	252	15.497
	Octadecanenitrile	265	0.229
	1-Dodecanol, 2-hexyl-	270	10.828
	1-Decanol, 2-octyl-	270	0.262
C_{20}	1,19-Eicosadiene	278	0.745
	Eicosane	282	0.334
		**Total**	**40.096**
C_{21-35}	Pentadecafluorooctanoic acid, tridecyl ester	596	5.019
	Oxalic acid, di(1-menthyl) ester	366	0.243
	Hexadecyl nonyl ether	368	0.520
	Octadecyl octyl ether	382	0.797
	Tetracosyl trifluoroacetate	450	0.253
	2-Methylhexacosane	380	0.374
	Tetracosyl pentafluoropropionate	500	0.254
	Hexacosyl trifluoroacetate	478	0.324
	Octacosyl trifluoroacetate	298	1.226
C_{36+}	Hexacosyl heptafluorobutyrate	578	0.256
	7,8-Epoxylanostan-11-ol, 3-acetoxy-	502	0.214

Bis(tridecyl) phthalate	530	1.361
Triacontyl heptafluorobutyrate	634	2.084
Dotriacontyl pentafluoropropionate	612	0.283
	**Total**	**13.207**
Dotriacontyl heptafluorobutyrate	662	1.475
Nonyl octacosyl ether	536	0.915
Tetratriacontyl pentafluoropropionate	640	2.087
Hexatriacontyl trifluoroacetate	618	5.363
Tetratriacontyl heptafluorobutyrate	690	1.752
Octatriacontyl trifluoroacetate	646	8.494
Tetrapentacontane, 1,54-dibromo-	914	1.560
Hexacontane	842	0.638
	**Total**	**22.285**

Table S3

Common Reaction Mechanisms and their Differential and Integral Rate expressions

Symbol	*Reaction Mechanism*	*Differential form, f(x)*	*Integral form, g(x)*
Reaction-order			
First order	F1	$(1-\alpha)$	$-\ln(1-\alpha)$
Second order	F2	$(1-\alpha)^2$	$(1-\alpha)^{-1}-1$
Third order	F3	$(1-\alpha)^3$	$½[(1-\alpha)^{-2}-1]$
n-th order	Fn	$(1-\alpha)^n$	$1/(n-1)[(1-\alpha)^{-(n-1)}-1]$
Diffusion			
One-way transport	D1	$½\,\alpha$	α^2
Two-way transport (Valensi)	D2	$[-\ln(1-\alpha)]^{-1}$	$\alpha+(1-\alpha)\ln(1-\alpha)$
Three-way transport (Jander)	D3	$2/3((1-\alpha)^{-2/3})[1-(1-\alpha)^{1/3}]^2$	$[1-(1-\alpha)^{1/3}]^2$
Three-way transport (Ginstling-Brounshtein)	D4	$2/3((1-\alpha)^{-1/3})-1]^{-1}$	$(1-2\alpha/3)-(1-\alpha)^{2/3}$
Geometrical contraction			
One dimension	R1	1	α
Two dimensions (contracting cylinder)	R2	$2(1-\alpha)^{1/2}$	$1-(1-\alpha)^{1/2}$
Two dimensions (contracting sphere)	R3	$3(1-\alpha)^{2/3}$	$1-(1-\alpha)^{1/3}$
Nucleation			
Two dimensional (Avrami-Erofeev)	A2	$2(1-\alpha)[-\ln(1-\alpha)]^{1/2}$	$[-\ln((1-\alpha)]^{1/2}$
Three dimensional (Avrami-Erofeev)	A3	$3(1-\alpha)[-\ln(1-\alpha)]^{2/3}$	$[-\ln((1-\alpha)]^{1/3}$
Exponential nucleation			
Power law, n = 1/2			
Power law, n = 1/3	P2	$1/2\alpha^2$	$\alpha^{1/2}$
Power law, n = 1/4	P3	$2/3\alpha^3$	$\alpha^{1/3}$
	P4	$3/4\alpha^4$	$\alpha^{1/4}$

[40]

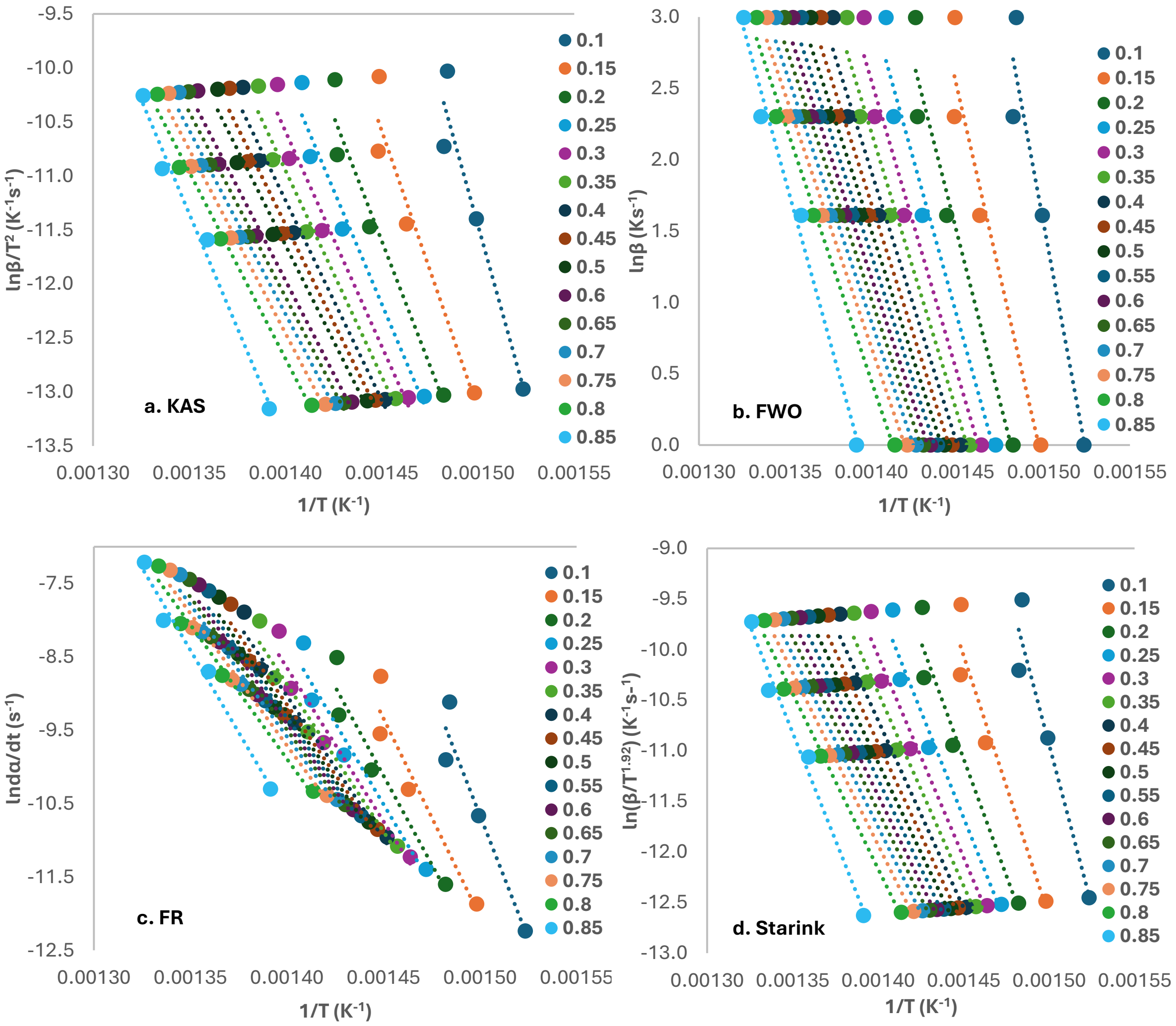

Figure. S4. *Linear plots for determination of Ea using* ***a.*** *KAS,* ***b.*** *FWO,* ***c.*** *FR, and* ***d.*** *Starink kinetic methods*

Table S4

Reaction Mechanisms and their Differential and Integral Rate expressions for conversions between 0.1 and 0.85

Model	1°C/min		5°C/min		10°C/min		20°C/min		Average		R^2
	E_a (kJ/mol)	lnA	E_a (kJ/mol)	lnA	E_a (kJ/mol)	lnA	E_a (kJ/mol)	lnA	E_a (kJ/mol)	lnA	
**F1**	198.07	31.0	170.50	26.7	162.84	25.6	145.56	23.2	169.24	26.62	0.9990
**F2**	269.12	44.1	228.18	37.1	218.81	35.7	195.54	32.2	227.91	37.27	0.9987
**F3**	355.74	60.0	297.99	49.7	286.64	47.7	256.04	43.0	299.10	50.09	0.9988
**D1**	300.61	48.0	264.51	41.8	252.14	39.7	226.56	35.8	260.95	41.33	0.9987
**D2**	330.91	52.9	289.74	45.6	276.54	43.4	248.44	39.1	286.41	45.25	0.9986
**D3**	368.04	58.2	320.30	49.6	306.13	47.2	274.93	42.3	317.35	49.33	0.9985
**D4**	343.16	53.7	299.84	45.9	286.32	43.6	257.20	39.1	296.63	45.59	0.9986

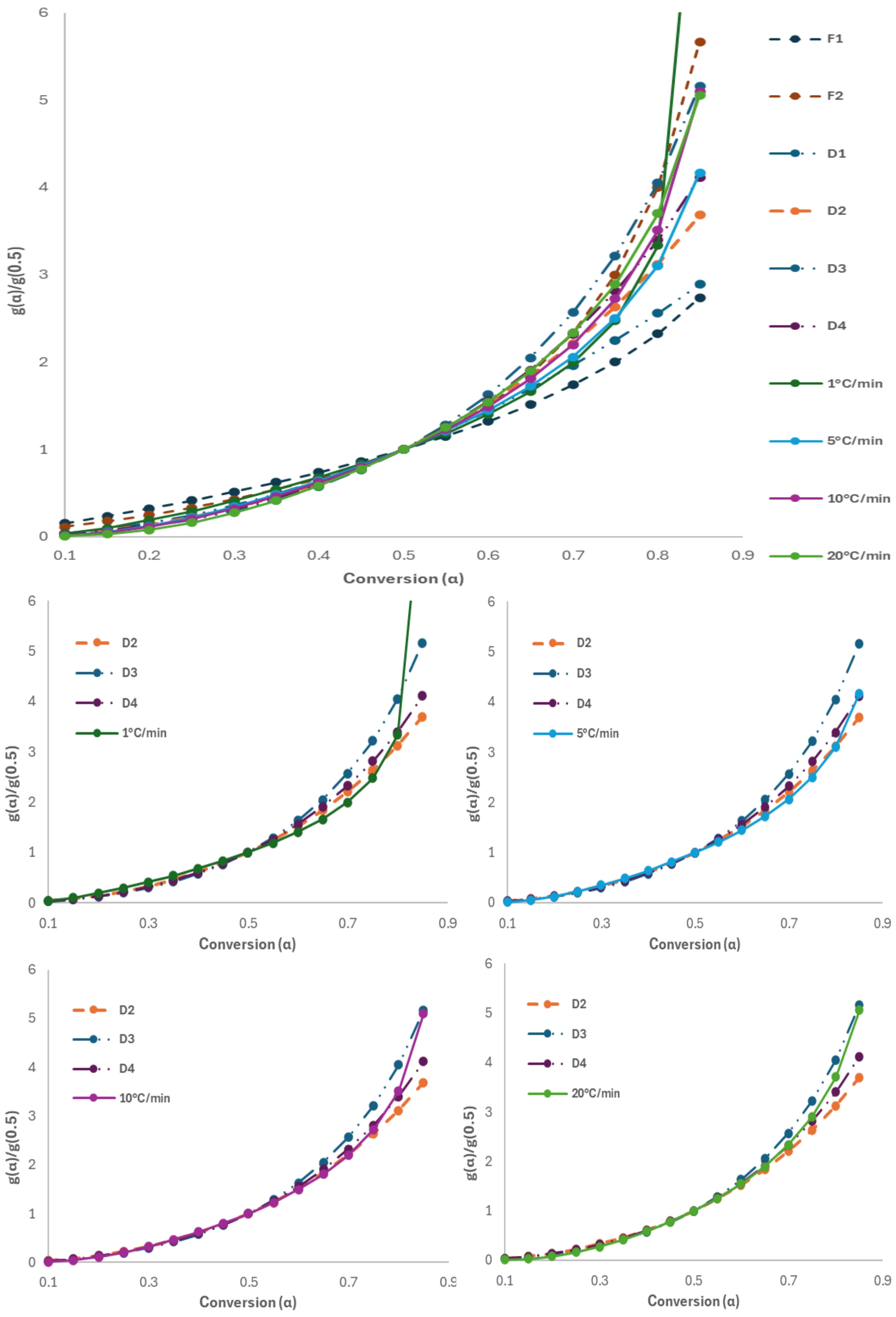

Figure. S5. *Comparison of theoretical and experimental master plots for full conversion range*

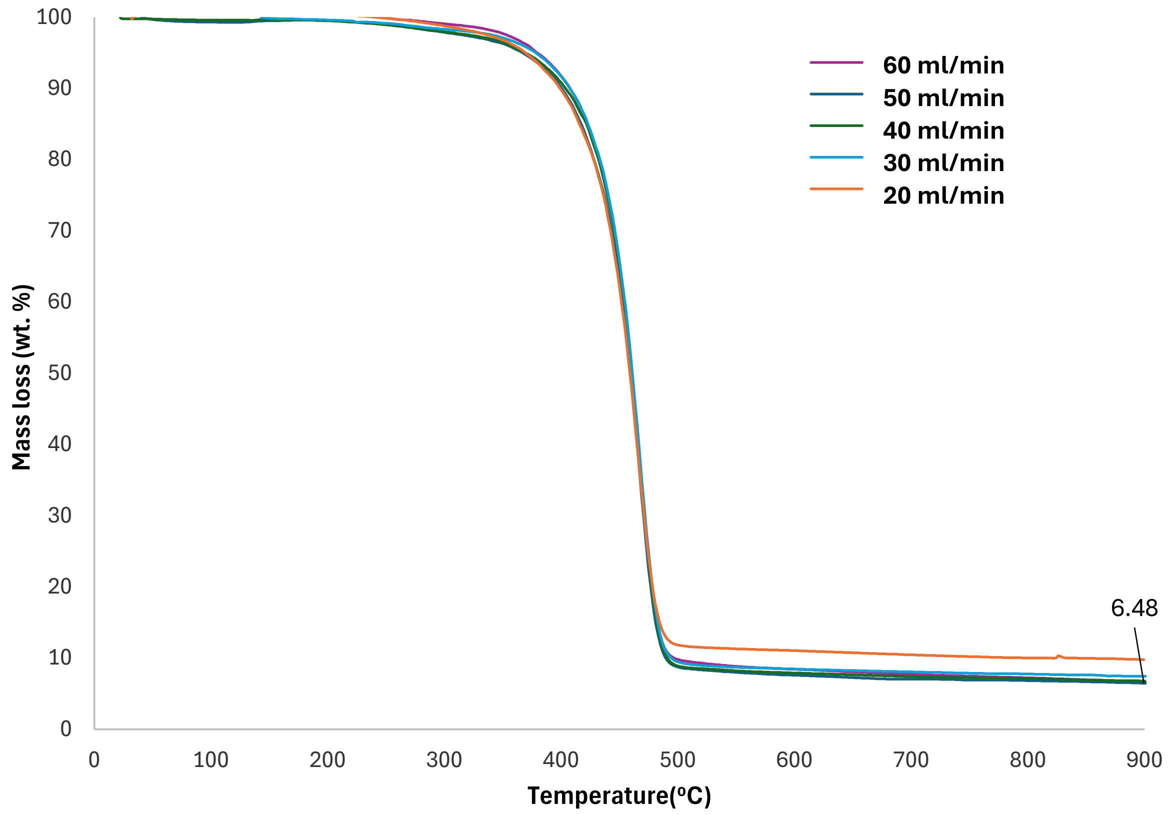

***Figure S6.* –** *N_2 flowrate comparison using 20°C/min heating rate*

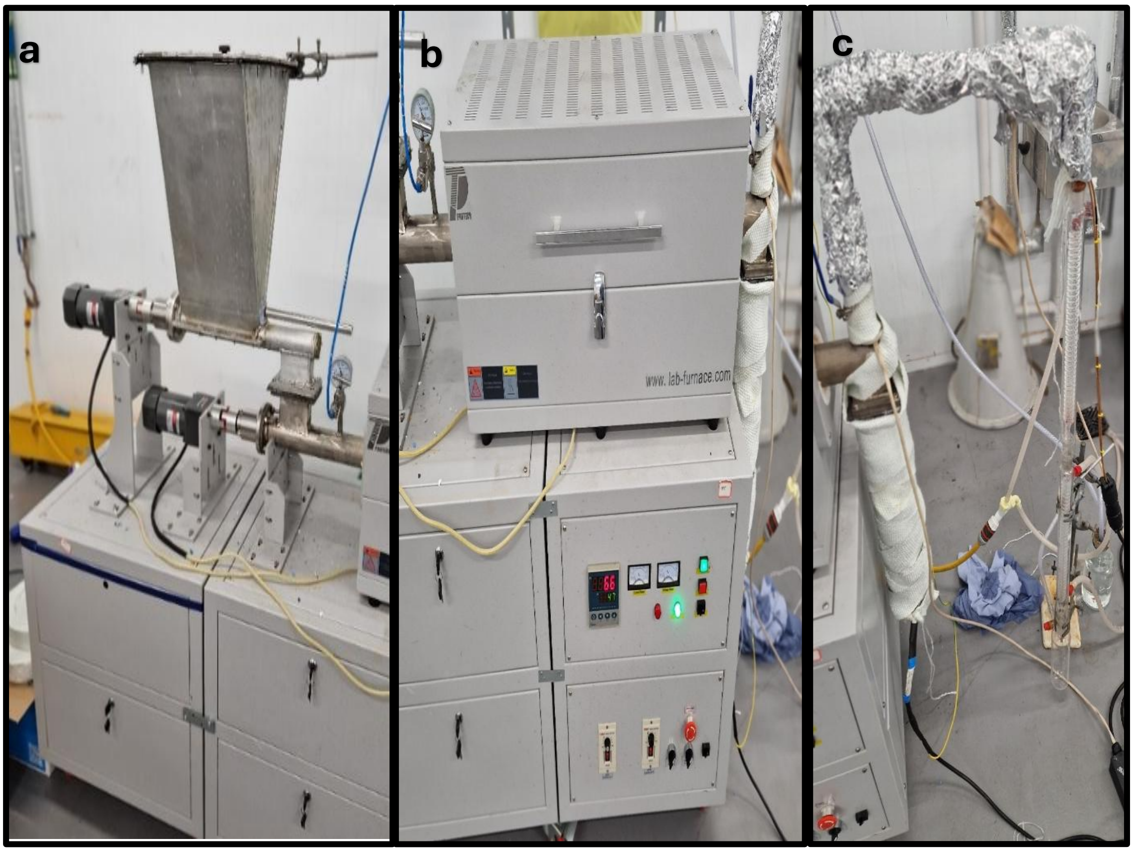

Figure S7a. *Hopper and feeding system,* ***b.*** *Auger reactor and furnace, and* ***c.*** *Condensers and sample bottle*
